# From radiomics to transformers in pancreatic cancer detection and prognosis

**DOI:** 10.3389/fmed.2025.1731922

**Published:** 2026-01-09

**Authors:** Maram Fahaad Almufareh, Samabia Tehsin, Mamoona Humayun, Sumaira Kausar, Asad Farooq, Haya Aldossary, Abeer Aljohani

**Affiliations:** 1 Department of Information Systems, College of Computer and Information Sciences, Jouf University, Sakaka, Saudi Arabia; 2 Centre of Excellence in Artificial Intelligence, Bahria University, Islamabad, Pakistan; 3 School of Computing, Engineering and the Built Environment, University of Roehampton, London, United Kingdom; 4 Computer Science Department, College of Science and Humanities, Imam Abdul Rahman Bin Faisal University, Jubail, Saudi Arabia; 5 Department of Computer Science and Information, Applied College, Taibah University, Madinah, Saudi Arabia

**Keywords:** attention, deep learning, early detection, multi-modal fusion, pancreatic ductal adenocarcinoma, radiomics, transformers

## Abstract

**Introduction:**

Pancreatic ductal adenocarcinoma (PDAC) remains one of the deadliest malignancies, primarily due to late diagnosis and poor therapeutic response. Advances in artificial intelligence (AI), particularly in medical imaging and multi-modal data integration, have created new opportunities for improving early detection and personalized prognostication.

**Methods:**

This systematic review was conducted according to the Preferred Reporting Items for Systematic Reviews and Meta-Analyses (PRISMA) 2020 statement. The protocol was prospectively registered with the Open Science Framework, covering studies published between 2015 and 2025.

**Results:**

Distinct from prior surveys that focus narrowly on specific algorithms or data types, this work introduces a generational taxonomy of AI approaches—ranging from classical radiomics-based machine learning to deep learning and contemporary transformer-based models—and maps their application to core clinical tasks such as detection, segmentation, classification, and outcome prediction. A key contribution is the integration of diverse datasets across imaging, pathology, and molecular sources; we further assess trends in availability, usage, and sample scale.

**Discussion:**

We critically evaluate limitations in generalizability, external validation, model calibration, and translational readiness, and outline recommendations for multi-center validation, standardized reporting, domain adaptation, and clinician-centered interpretability.

**Systematic review registration:**

https://doi.org/10.17605/OSF.IO/2DVHJ.

## 1 Introduction

**Figure 1:**
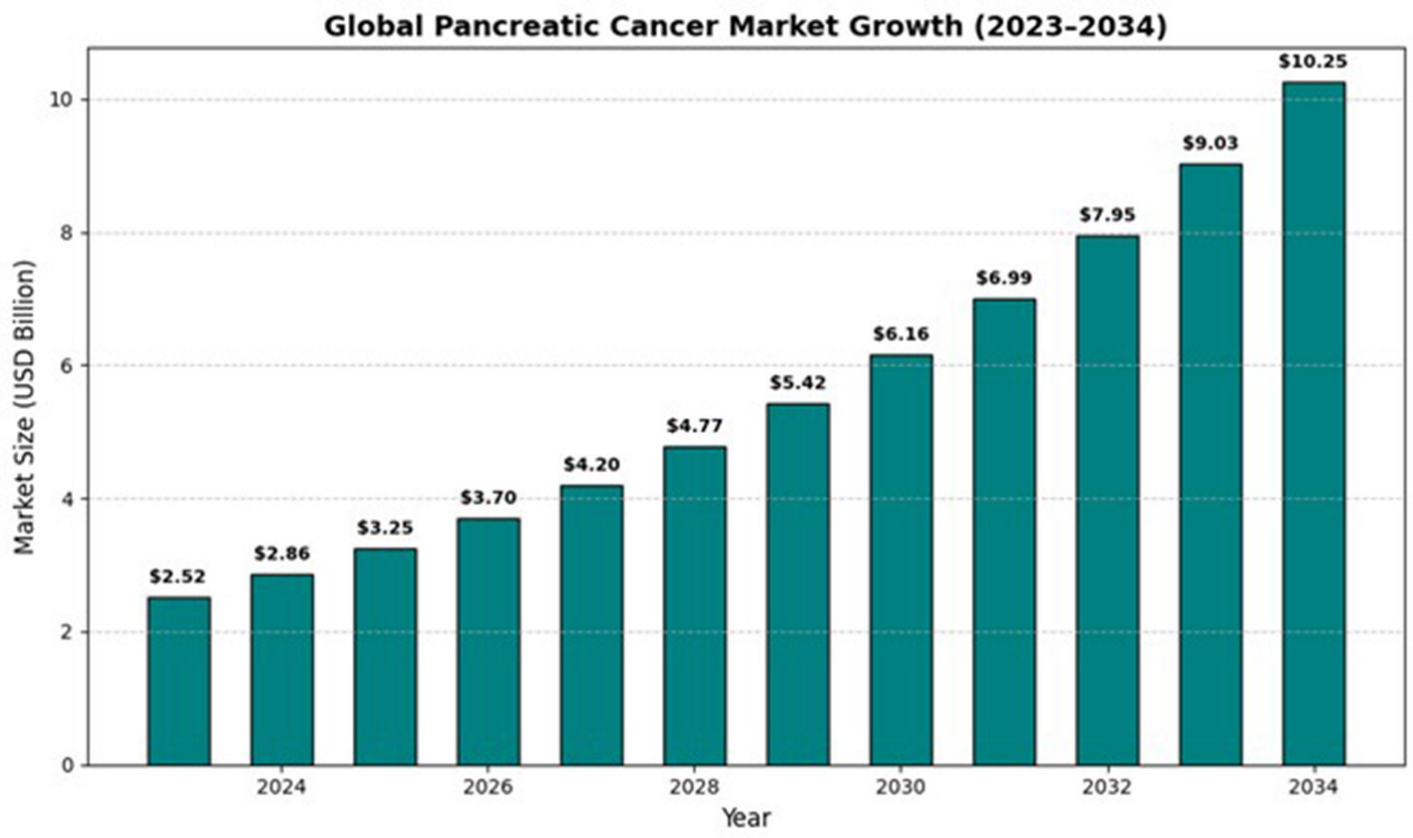
Projected global pancreatic cancer market growth, 2023–2034 (Precedence Research, 2024). The market is expected to rise from approximately United States Dollar (USD) 2.86 billion in 2024 to USD 10.25 billion by 2034 Compound Annual Growth Rate (CAGR) ≈ 13.62%, reflecting expanding investment in diagnostics, therapeutics and clinical research that may accelerate translation of Artificial Intelligence-enabled detection and prognosis systems.

Cancer remains one of the world's most formidable public-health challenges. The Global Cancer Observatory reported almost 20 million new diagnoses and 9.7 million deaths in 2022 (Figure 1), translating to an economic burden that exceeds 1% of global gross domestic product each year (1). Recent market reports reflect the growing clinical interest and research activity in pancreatic cancer. Precedence Research (2024) forecasts strong expansion of the pancreatic cancer market through 2034 driven by rising investment in diagnostics (2), therapeutics and clinical trials. This commercial growth both supports and mirrors the increased funding for AI based detection and prognostic technologies that are the main focus of this review.

Despite incremental gains in screening and therapy, the absolute number of cancer-related fatalities continues to rise with population aging and growth (3). Pancreatic cancer (PC), of which approximately 90% are pancreatic ductal adenocarcinomas (PDAC), deserves special attention because its mortality far exceeds its incidence ranking. Globally it is the twelfth most commonly diagnosed malignancy, yet it already ranks seventh among cancer deaths (3, 4). Five-year relative survival in high-income countries has only recently crept into double digits—roughly 11%–13%—making PDAC the deadliest of the major solid tumors (5). Contributing factors include a stealthy symptom profile, an anatomically concealed primary site and a dense desmoplastic micro-environment that confers intrinsic resistance to cytotoxic therapies (3). Without intervention, PDAC is projected to become the second leading cause of cancer mortality in North America and parts of Europe by 2030 (4).

The global burden of pancreatic cancer has continued to rise over the past three decades. According to GLOBOCAN 2022, there were 510,992 new cases worldwide (1). Estimates from the Global Burden of Disease (GBD) 2019–2021 update indicate incident cases increased from about 489,862 in 2019 to 508,533 in 2021, while deaths rose from 486,869 to 505,752 over the same period (3). The age-standardized incidence rate (ASIR) decreased slightly from 6.04 to 5.96 per 100,000, whereas the age-standardized death rate (ASDR) declined from 6.03 to 5.95 per 100,000 (3). These aggregate figures mask striking geographic disparities. High-income regions such as North America, Western Europe and high-income Asia–Pacific have ASIRs approaching 10 per 100,000, whereas low-SDI countries have rates as low as 1.6 per 100,000 (3). Case fatality mirrors incidence because PDAC is frequently lethal once symptomatic. The incidence-to-mortality ratio was only ≈1.28 in 2024, and more than 85% of patients are diagnosed at an unresectable or metastatic stage (3).

Table 1 summarizes recent estimates from high-incidence regions. In the United States, the National Cancer Institute projects 67,440 new cases and 51,980 deaths for 2025. Although pancreatic cancer will account for only 3.3% of new malignancies, it will cause 8.4% of all cancer deaths (5). The United States has seen incidence climb by about 0.7% per year since 2001, and PDAC is forecast to become the second leading cause of cancer death by 2030 (4). European countries such as Hungary, the Czech Republic and Finland report age-standardized mortality rates exceeding 8 per 100,000 (4). In Asia, China already accounts for over 25% of worldwide PDAC deaths, and Japan has experienced one of the steepest rises in incidence among high-income countries (3). The median age at diagnosis is approximately 70 years, and more than 80% of patients present with unresectable or metastatic disease (3).

**Table 1:** Recent regional estimates for pancreatic cancer incidence and mortality.

**Region**	**Epidemiological statistics**	**Notable findings**
**Global (2021–2022)**	510,992 new cases in 2022; 508,533 cases and 505,752 deaths in 2021; ASIR decreased from 6.04 to 5.96 per 100,000 (1, 3)	Slight decline in age-standardized rates but absolute numbers rising; incidence-to-mortality ratio ≈1.28.
**United States (2025)**	67,440 new cases and 51,980 deaths projected; pancreatic cancer accounts for 3.3% of new cancers but 8.4% of cancer deaths (5)	Incidence rising at ≈0.7% annually; PDAC projected to be the second leading cause of cancer death by 2030 (4).
**Europe (2024)**	Hungary, Czechia and Finland have age-standardized mortality >8 per 100,000 (4)	Mortality rates in some European nations are among the highest globally.
**Asia (2024)**	China accounts for >25% of PDAC deaths; Japan reports one of the steepest rises in incidence among high-income countries (3)	Demographic shifts and rapid population aging drive a large share of the global burden.

Socio-economic inequalities magnify these differences. A recent analysis using GBD 2021 data showed that countries with high SDI had ASIRs around 10 per 100,000 and ASDRs near 9.4 per 100,000, compared with rates around 1.6–1.7 per 100,000 in low-SDI countries (3). The number of disability-adjusted life-years (DALYs) attributable to pancreatic cancer rose from 10.9 million in 2019 to 11.3 million in 2021, highlighting the growing societal and economic toll (3). Projection models estimate that incident cases and deaths will both exceed 875,000 by 2044 (3).

Pancreatic carcinogenesis is multifactorial, with both modifiable and non-modifiable determinants. Modifiable lifestyle factors include tobacco smoking, obesity, diabetes, diet and alcohol consumption. Smoking remains the strongest environmental risk factor: pooled analyses show that current smokers have nearly a two-fold increase in risk compared with never smokers, and heavy smokers (>25 cigarettes day^−1^) can experience a 2.7-fold elevation (6). Risk declines after cessation but may not return to baseline until 15–20 years later (6). A meta-analysis reported that current smokers have a 75% increased risk of pancreatic cancer relative to never smokers and that elevated risk persists for at least a decade after quitting (6). Second-hand smoke exposure appears to play a minor role (6).

Obesity and metabolic dysfunction are increasingly recognized contributors. A pooled analysis of cohort studies found that obesity approximately doubles the risk of pancreatic cancer in both men and women, and each 5 kg m^−2^ increase in body-mass index raises risk by about 12% (6). Adipose tissue produces pro-inflammatory cytokines and increases insulin resistance, creating a carcinogenic milieu. Type 2 diabetes mellitus (T2DM) is both a risk factor and a consequence of pancreatic cancer: long-standing T2DM increases PDAC risk by 1.5–2.0-fold, whereas new-onset diabetes confers a 5–8-fold increased risk within one to three years (6). Indeed, a large proportion of patients with pancreatic cancer have diabetes or impaired glucose tolerance at diagnosis (6). Other dietary and lifestyle factors—such as heavy alcohol consumption, diets rich in red and processed meats, low intake of fruits and vegetables, and chronic pancreatitis—have also been implicated, although effect sizes are generally smaller.

Non-modifiable factors include age, sex, ethnicity and genetic predisposition. Incidence increases dramatically with age and peaks between 70 and 74 years (3). Males tend to have higher age-standardized incidence and mortality rates than females across all age groups (3). Familial pancreatic cancer accounts for 5–10% of cases; germline mutations in *BRCA1/2, CDKN2A, PALB2, STK11/LKB1, TP53* and mismatch-repair genes confer markedly elevated lifetime risks. Hereditary pancreatitis (*PRSS1* mutations), Peutz–Jeghers syndrome and familial atypical multiple mole melanoma syndrome are notable syndromes requiring surveillance. Pancreatic cancer risk also varies by ethnicity; for example, African-American populations in the United States experience incidence and mortality rates about 30% higher than those of Caucasian populations, likely reflecting a combination of genetic, metabolic and socio-economic factors (3).

Early detection remains the cornerstone for improving pancreatic-cancer outcomes. However, current screening modalities lack sensitivity for precursor lesions, and the overall rarity of PDAC precludes population-wide screening. Standard imaging techniques such as abdominal ultrasound and computed tomography have limited ability to detect small pancreatic tumors. Pre-diagnostic CT(Computed Tomography) scans often fail to reveal abnormalities in more than half of patients, and subtle signs may precede the clinical diagnosis by 3–36 months (7). Consequently, only about 13.6% of PDAC cases are diagnosed while still localized, and over 85% present with locally advanced or metastatic disease (3). When patients are diagnosed at an early stage and can undergo complete resection followed by multi-modal therapy, median overall survival can exceed 60 months (7).

Given the low prevalence of PDAC in the general population, surveillance strategies are currently recommended only for high-risk individuals—those with strong family histories or pathogenic germline mutations. Ongoing prospective cohorts (for example, the CAPS and Dutch familial pancreatic cancer studies) monitor high-risk participants with annual magnetic resonance imaging (MRI) and endoscopic ultrasound (EUS). A recent update involving approximately 1,700 participants with familial or genetic risk factors reported that surveillance detected tumors at an earlier stage: 38.5% of screen-detected cancers were stage I compared with 10.3% in the general population; 5-year survival reached 50% among the surveillance cohort vs. 9% for non-screened patients (8). The study underscores the potential of targeted screening to extend survival but also highlights logistical challenges: surveillance requires specialized centers with multidisciplinary expertise, and false-positive results can lead to unnecessary interventions (8).

The advent of artificial intelligence and machine-learning techniques offers hope for earlier detection and more accurate risk stratification (9). Radiomics extracts high-dimensional quantitative features from imaging data, capturing subtle textural and morphological patterns that are imperceptible to the human eye. Deep-learning architectures—particularly convolutional neural networks (CNNs) and U-Net–based segmentation models—have demonstrated promising performance in identifying pancreatic lesions and classifying intraductal papillary mucinous neoplasms (IPMNs). A recent systematic review of AI-based IPMN imaging reported classification accuracies ranging from 60% to 99.6%, although heterogeneity in study populations, imaging protocols and analytic pipelines limits direct comparison (7, 10). Most studies relied on CT data despite guidelines favoring MRI, and many used small, single-center cohorts without external validation, leading to high risk of bias (7). Standardized frameworks, large multi-institutional datasets and rigorous external validation are urgently needed.

AI-augmented imaging may also facilitate detection of subtle pre-diagnostic changes. For example, radiomics and deep-learning models can segment the pancreas automatically and identify textural or shape alterations months to years before clinical presentation. In a recent review of AI-augmented imaging, radiomic signatures from routine CT were able to identify early changes that preceded diagnosis by 3–36 months (7). Such models, once validated and integrated into clinical workflows, could trigger further evaluation or enrolment into high-risk surveillance programmes. Nevertheless, adoption of AI in clinical practice raises issues of data privacy, algorithmic fairness, interpretability and regulatory oversight (7, 10).

Table 2 summarizes seven recent AI-focused reviews on pancreatic cancer. While these reviews document progress in CNNs, transformers, and radiomics, they consistently reveal gaps in comprehensive attention mechanism surveys, unified dataset visualizations and external validation strategies—limitations that our paper addresses.

**Table 2:** Summary of recent AI-focused reviews on pancreatic cancer (2023–2025).

**References**	**Journal/Title**	**Scope**	**Advantages**	**Limitations**
Podĭnă et al. (11)	Artificial Intelligence in Pancreatic Imaging: A Systematic Review	Systematic review of AI in pancreatic imaging (CT/MRI/EUS)	PRISMA-style search; clear overview of CNN/radiomics applications and clinical use cases	Imaging-only scope; limited attention/transformer mapping; no comprehensive dataset landscape/visualizations
Yao et al. (12)	Deep learning and radiomics approaches for pancreatic cancer diagnosis from medical imaging	Narrative review focused on CT/MRI CAD with DL & radiomics	Summarizes CNNs; mentions transformers; provides workflow/metric diagrams	Imaging-only; brief attention/transformer coverage; lacks broad dataset comparisons/visualizations
Mishra et al. (13)	ML Models for Pancreatic Cancer Risk Prediction Using EHR—Systematic Review and Assessment	Systematic review of EHR-based risk models	Methodological appraisal; figures on model types and validation	EHR-only; models mostly logistic regression; no attention/transformer landscape; no dataset visualizations
Qadir et al. (7)	AI in IPMN Imaging: A Systematic Review	Systematic review on IPMN (cyst) imaging	Breakdown by modality and stage of translation; PRISMA and study distribution figures	Narrow to IPMN & imaging; small single-center studies; few prospective evaluations; minimal EUS; limited attention coverage; limited dataset visualizations
Zhang et al. (14)	Effectiveness of Radiomics-Based ML for PDAC vs. Mass-Forming Pancreatitis: Systematic Review & Meta-analysis	Diagnostic performance meta-analysis (primarily radiomics)	Pooled sensitivity/ specificity; subgroup analyses	Single task focus; moderate methodological quality;minimal attention/transformer coverage; no cross-modality dataset landscape/visualizations
**References**	**Journal/Title**	**Scope**	**Advantages**	**Limitations (gaps our paper covers)**
Antony et al. (10)	AI-Augmented Imaging for Early PDAC Detection	Narrative review of CT-centric AI for early PDAC	Highlights pre-diagnostic detection and segmentation; states barriers clearly	CT-focused; lacks comprehensive attention/transformer survey; minimal coverage of non-imaging modalities, or dataset visualizations
Yu et al. (84)	Combining Multimodal Medical Imaging and AI for Early Diagnosis of Pancreatic Cancer	Perspective/review advocating for multimodal imaging fusion	Articulates need for multimodal fusion; summarizes imaging performance and interpretability issues	Opinion piece; acknowledges current research is single-modality; does not catalog attention/transformers; no broad dataset visualizations

While recent reviews organize AI studies by **imaging modality** [Podina et al.(11), Yao et al.(12)], **clinical task** (detection, segmentation, classification), **data type** [EHR-only: Mishra et al.(13)], or **clinical subtype** [IPMN: qadir et al.(7); PDAC vs. pancreatitis: Zhang et al.(14)]—approaches that effectively catalog method–modality pairings for specific protocols—our **generational taxonomy** provides a distinct meta-level view by organizing studies into three methodological waves: conventional machine-learning/radiomics pipelines (Generation 1, 2015–2020), deep-learning CNNs (Generation 2, 2017–2023), and attention/transformer-based multi-modal fusion (Generation 3, 2020–present). This temporal framework enables us to **quantify performance evolution** [AUC 0.84–0.98 in Generation 1 → 0.92–0.99 in Generation 2 → 0.996 in Generation 3 (15); segmentation Dice 0.19–0.70 → 0.57–0.87], synthesize findings **across all modalities** (CT/MRI, histopathology, genomics, biomarkers) within a single coherent framework, reveal **validation trends** (external validation 50% → 57% → 67% across generations; multi-center validation 0% → 29% → 22%), and identify the **research frontier** (attention-augmented, domain-adaptive architectures) by extrapolating from the architectural trajectory—advantages not accessible through modality-specific or task-specific organization. As detailed in Table 2, this generational perspective complements existing reviews by revealing **temporal, cross-modality, and performance-evolution patterns** that become visible only when viewing AI methods as successive generations building on architectural innovations.

**Figure 2:**
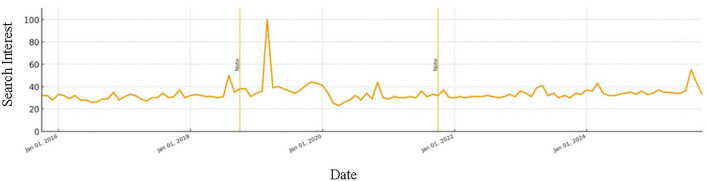
Global Google Trends interest for “Artificial Intelligence,” 2015–2025 (Google Trends, 2025).

Figure 2 illustrates global search interest in “Artificial Intelligence” from 2015 to 2025, highlighting sharp public attention peaks that coincide with major AI advances (16). Such public and media surges often track research investment and adoption cycles that accelerate translation of AI techniques into healthcare applications.

This review makes several unique contributions to the growing literature on AI-enabled pancreatic cancer detection and prognosis. First, it conducts a rigorous and reproducible search across PubMed and Google Scholar covering 2015–2025 to capture more than sixty peer-reviewed studies that apply machine learning, deep learning or attention-based methods to imaging, histopathology and molecular data. The search strategy reduces the risk of missed early-stage or conference works by including theses and preprints in addition to indexed journals.

- **Comprehensive literature scope:** Unlike prior reviews that focus exclusively on electronic health record risk models or histopathology, we synthesize results from multi-modal data sources—radiomics, CT/MRI imaging, endoscopic ultrasound, histopathology, genomics, and proteomics—spanning more than 60 studies published between 2015 and 2025.
- **Structured classification of methods and tasks:** We introduce a clear taxonomy that groups AI approaches into three methodological generations—classical machine-learning/radiomic pipelines, deep neural networks and attention- or transformer-enhanced architectures—and map them to clinical tasks (detection, segmentation, classification/subtyping, and prognosis). This framework facilitates cross-comparison of algorithmic advances and reveals trends across modalities.
- **Critical appraisal of methodological quality:** Beyond reporting accuracy, AUROC, and Dice scores, we assess whether studies used patient-level splits, external validation cohorts and proper prevalence reporting. Our synthesis highlights that many models are retrospective and single-center with limited robustness, underscoring the need for prospective, multi-center evaluation.
- **Evidence for attention and multi-modal fusion:** We show that attention-augmented and transformer-based models achieve consistent improvements in diagnostic accuracy and segmentation performance across tasks. By fusing imaging with biomarkers and clinical variables, multi-modal networks outperform single-modality baselines, demonstrating a path toward earlier, non-invasive detection.
- **Identifying gaps and future research directions:** Our review discusses issues rarely addressed in previous surveys—domain shift, algorithmic fairness, data governance and integration into clinical workflows—and proposes a forward-looking research agenda. Recommendations include conducting prospective trials, adopting common reporting standards, investing in domain generalization and federated learning, and co-designing interpretable algorithms with clinicians.
- **Bridging disparate literatures:** By contextualizing machine-learning advances alongside epidemiological and risk-factor data and comparing AI methods across modalities, we provide a holistic understanding of how AI can support early pancreatic cancer detection and personalized management. This integrative perspective is largely missing from existing domain-specific reviews.

This set of contributions positions our work as a comprehensive, methodologically rigorous and forward-looking synthesis that highlights both the promise of attention-based, multi-modal AI and the steps required for safe and equitable clinical translation.

**Figure 3:**
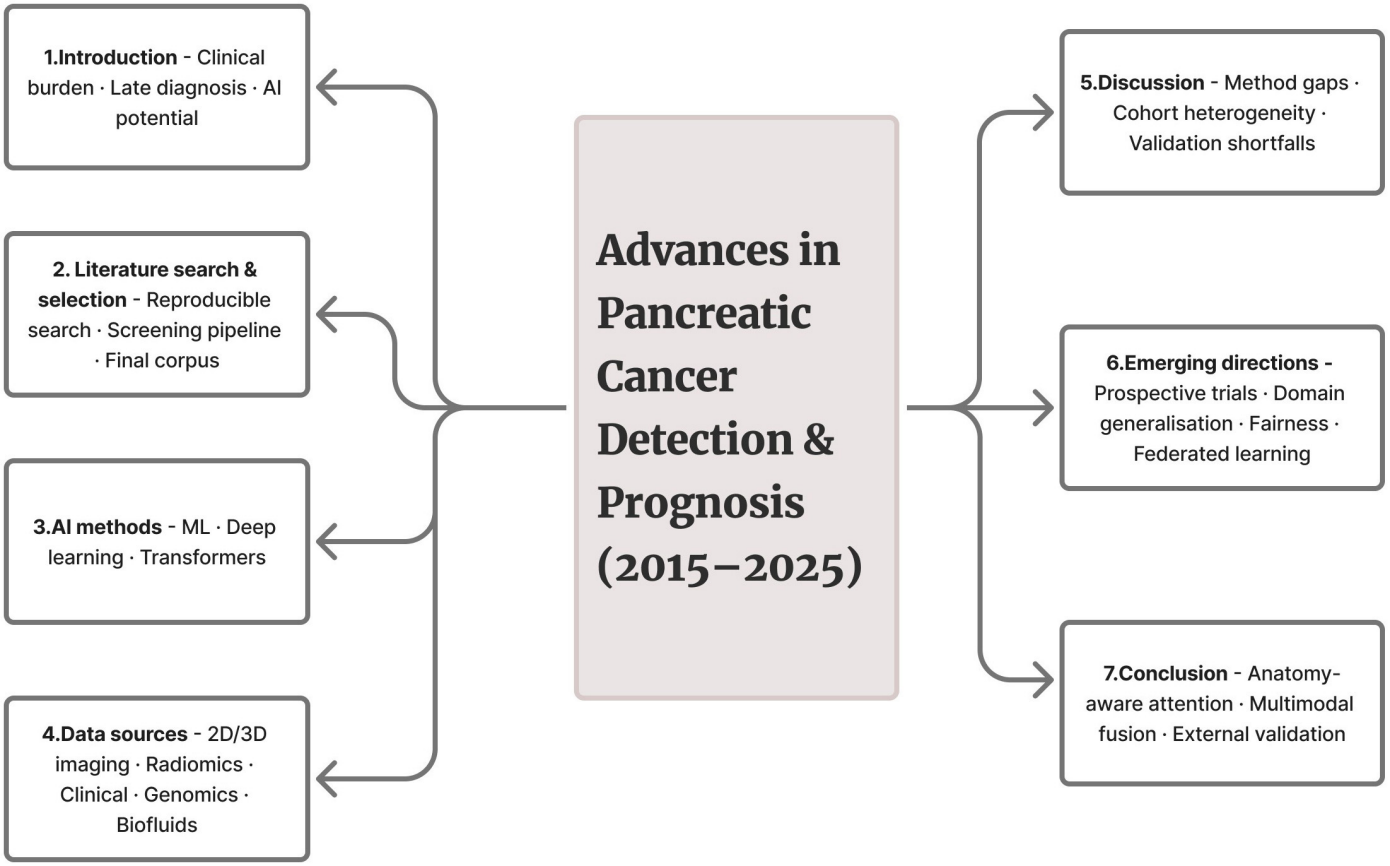
Schematic overview of the review framework outlining the progression from clinical motivation to emerging Artificial Intelligence research directions in pancreatic cancer detection and prognosis (2015–2025).

This review is organized to walk the reader from clinical motivation to actionable research priorities (Figure 3): Section 1 provides the clinical and epidemiological background (incidence, mortality, key risk factors) and motivates the need for improved surveillance and AI-augmented detection, with focused subparts on risk factors, surveillance, and the role of emerging AI methods; Section 2 details the literature search and selection strategy (databases, search queries, deduplication, title/abstract triage, inclusion/exclusion criteria, full-text review and synthesis protocol) so readers can reproduce the corpus assembly; Section 3 synthesizes the surveyed AI work by methodological generation—Section 3.1 conventional machine-learning and radiomics pipelines, Section 3.2 deep-learning models for detection/segmentation/classification, and Section 3.3 attention- and transformer-based architectures and multi-modal fusion—highlighting representative studies and performance patterns; Section 4 catalogs the data sources that underpin the field (Section 4.1 two-dimensional imaging, Section 4.2 three-dimensional CT/MRI volumes, Section 4.3 radiomics, Section 4.4 clinical/registry data, Section 4.5 genomic/molecular assays, and Section 4.6 biofluid/biomarker panels) and links data regimes to suitable model classes; Section 5 discusses cross-cutting issues, methodological gaps, and interpretation (e.g., patient-level splitting, external validation, and domain shift); Section 6 outlines emerging directions and concrete recommendations (prospective trials, reporting standards, domain generalization, fairness, and federated learning); and Section 7 concludes with priorities for clinical translation.

## 2 Literature search and selection strategy

**Figure 4:**
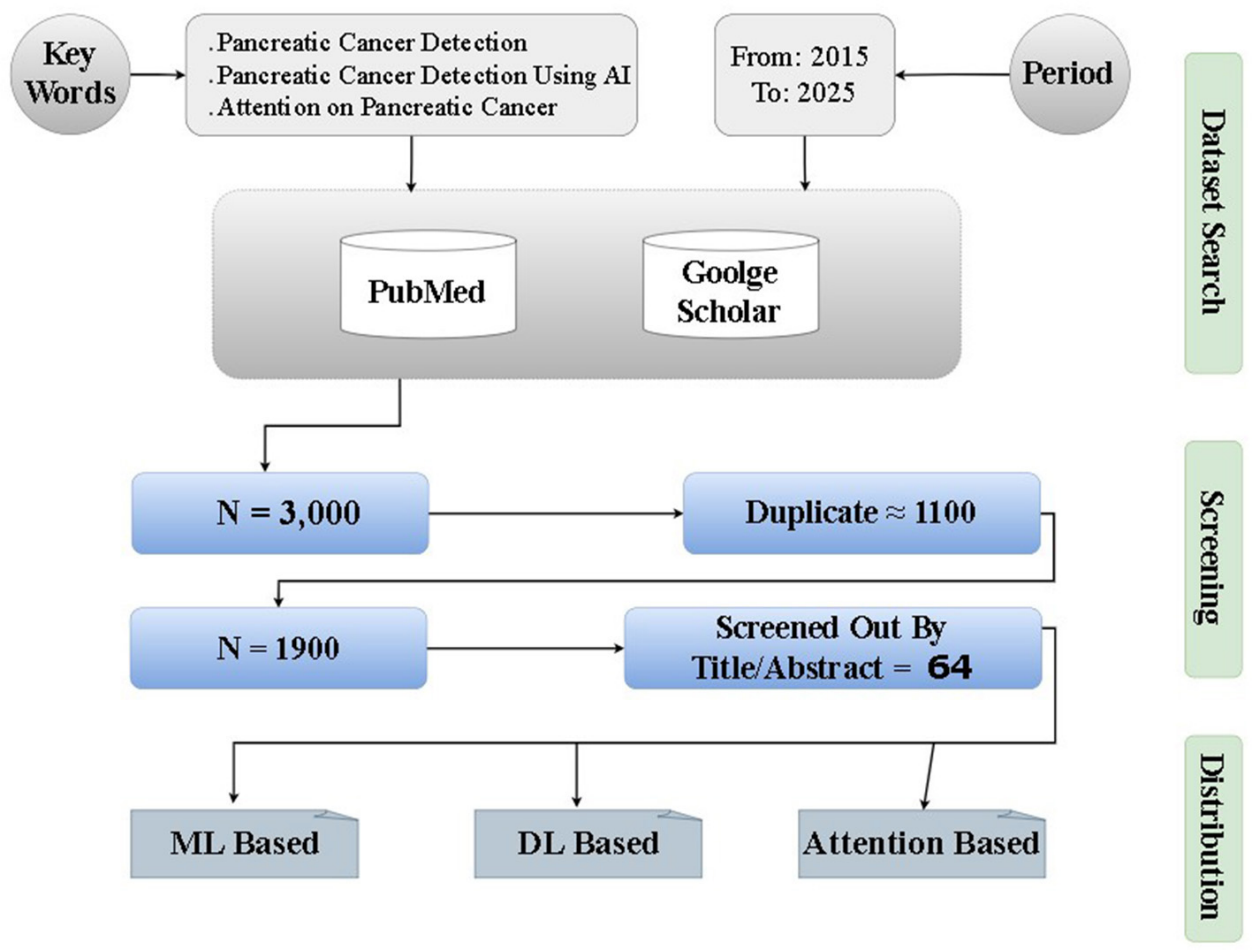
Preferred Reporting Items for Systematic Reviews and Meta-Analyses (PRISMA)-style flow diagram summarizing the study selection process for this review.

We performed a structured, reproducible literature search and selection procedure to identify primary studies that applied machine-learning (ML), deep-learning (DL), or attention-based methods to pancreatic cancer imaging, pathology or molecular data. The goal was to capture methods that directly address detection, segmentation, classification or prognostication in pancreatic disease using computational approaches. The overall workflow is illustrated in Figure 4.

This systematic review was conducted according to the Preferred Reporting Items for Systematic Reviews and Meta-Analyses (PRISMA) 2020 statement. The protocol was prospectively registered with [Open Science Framework (OSF), OID: https://doi.org/10.17605/OSF.IO/2DVHJ ].

### 2.1 Data sources and search strategy

Searches were run in PubMed and Google Scholar for the period 2015–2025. We selected these two resources because PubMed provides comprehensive coverage of biomedical journal literature and indexing (including MEDLINE), while Google Scholar expands coverage to conference proceedings, theses, preprints, and other scholarly material that may report novel computational methods not yet indexed in PubMed. The combined use reduces the chance of missing relevant methodological or early-stage engineering reports.

The search combined keywords and simple Boolean logic to balance sensitivity and precision. Example search queries used (adapted to each database syntax) were:


  (“Pancreatic Cancer Detection” OR
  “Pancreatic Cancer Detection Using AI” OR
  “Attention on Pancreatic Cancer”)


Searches were restricted to records published between 2015 and 2025 (inclusive). No language restriction was applied at the search stage, but non-English records were screened on title/abstract and translated where necessary for eligibility assessment.

### 2.2 Deduplication and initial yield

The initial combined search returned approximately 3,000 records. We performed automatic deduplication using reference-management software followed by manual inspection; roughly 1,100 duplicate records were removed, leaving about 1,900 unique records for title and abstract screening.

### 2.3 Title and abstract screening

Title and abstract screening was adopted as the principal triage step to exclude clearly irrelevant records prior to full-text review. This approach was necessary because the literature volume is large and many records that mention “pancreatic cancer” are peripheral (for example, biomarker discovery, basic biology, or therapeutic studies) rather than studies that develop or evaluate computational detection or image-based models; screening titles and abstracts therefore provides an efficient way to prioritize papers that explicitly describe ML/DL/attention methods or algorithmic evaluation. Equally important, title/abstract screening increases methodological focus: many biomedical papers use AI-related terms in passing, and the abstract is usually the first reliable source for whether a paper reports model architectures, training data, evaluation metrics or genuine algorithmic contributions rather than superficial references. Finally, documenting and applying explicit title/abstract criteria improves practical reproducibility by making the triage decisions auditable and repeatable. Two reviewers independently screened all titles and abstracts against the pre-specified eligibility criteria; disagreements were resolved through discussion and, where necessary, by arbitration from a senior reviewer. Following this procedure the corpus was reduced to 100 studies selected for full-text evaluation.

### 2.4 Inclusion and exclusion criteria

#### 2.4.1 Inclusion criteria

1. Studies that apply ML, DL, or attention-based methods to pancreatic cancer imaging, histopathology, or molecular data for tasks such as detection, segmentation, classification, subtype discrimination or prognostication.
2. Original research articles reporting methods and evaluation (i.e., not purely review articles or opinion pieces).
3. Studies that report quantitative performance metrics (e.g., accuracy, AUROC, Dice, sensitivity/specificity) on defined datasets.

#### 2.4.2 Exclusion criteria

1. Purely biological or wet-lab studies without computational model development or evaluation.
2. Reviews, editorials, commentaries, perspective pieces and protocols without primary experimental results.
3. Papers that do not provide enough methodological detail to interpret the model (for example, a short abstract only with no methods or results).

### 2.5 Full-text review and final selection

Full texts of the 64 candidate studies were retrieved and assessed in detail. During this stage we confirmed that the studies (i) presented sufficient methodological detail (data sources, preprocessing, model architecture, training, and validation strategy), (ii) used appropriate evaluation procedures (e.g., train/test splits, cross-validation, patient-level separation where applicable), and (iii) reported metrics relevant to the tasks claimed. Studies that failed to meet these standards or were duplicative (e.g., extended conference abstract later published as a journal article) were excluded. The final included set for synthesis is described in Section 3 and in Table 3.

**Table 3:** Comprehensive methodological quality analysis: validation strategies across model generations.

**Model generation**	**Single-center internal only**	**Multi-center studies**	**External validation**	**Performance metrics**
**Conventional ML (8 studies)**				
Internal validation only	3/8 (37.5%)	—	0/8	—
Single-center + external	4/8 (50%)	—	4/8	AUC 0.84–0.98
Large-scale/Registry	1/8 (12.5%)	—	—	99.97% acc.
**Subtotal:**	**3/8 internal**	**0/8 (0%)**	**4/8 (50%)**	
*Notable studies:*	(26) (ext. AUC 0.98); (22) (EHR 29,230 cases, AUC 0.84);			
	PancRISK (ext. AUC 0.94); SEER registry (99.97% acc.)			
**Deep learning (7 studies)**				
Internal validation only	3/7 (43%)	—	0/7	Acc. 99.8%
Single-center + external	2/7 (29%)	—	2/7	AUC 0.95–0.99
Multi-center + external	2/7 (29%)	2/7 (29%)	2/7	AUC 0.92
**Subtotal:**	**3/7 internal**	**2/7 (29%)**	**4/7 (57%)**	
*Notable studies:*	(46) (Taiwan+US multi-center, ext. AUC 0.92); chen2023 (nationwide ext. AUC 0.95);			
	viriyasaranon2023 (Korean multi-center, **performance drop 94.3% → 82.5%**)			
**Attention/transformer (9 studies)**				
Internal validation only	3/9 (33%)	—	0/9	Acc. 92%–94%
Single-center + external	4/9 (44%)	—	4/9	Dice 0.80–0.87
Multi-center + external	2/9 (22%)	2/9 (22%)	2/9	AUC 0.96–0.99
**Subtotal:**	**3/9 internal**	**2/9 (22%)**	**6/9 (67%)**	
*Notable studies:*	PANDA (**9-center study**, ext. AUC 0.987 lesion detection, 0.984 across 9 external sites);			
	DA-TransUNet (6 datasets validation); (77) (multi-center LNM, AUC 0.83)			
**Overall (24 studies)**				
Single-center, internal only	9/24 (37.5%)	—	—	—
Single-center + external val.	10/24 (41.7%)	—	10/24	—
Multi-center studies	—	4/24 (16.7%)	—	—
External validation (total)	—	—	14/24 (58.3%)	—
**Summary:**	**37.5% internal**	**16.7% multi-ctr**	**58.3% external**	

**Key findings from comprehensive analysis:**

• 37.5% of studies relied exclusively on single-center, internal-only validation, risking overfitting to local protocols.

•Only 16.7% employed multi-center designs, limiting assessment of cross-institutional generalizability.

• 58.3% reported external validation, with Attention/Transformer models showing highest adoption (67%).

•Multi-center studies documented performance degradation: (60) showed **11.8% accuracy drop** (94.3% → 82.5%) from internal Korean cohort to external U.S. validation, confirming domain shift effects.

•PANDA (9-center study) maintained strong performance (AUC 0.984–0.987) through large-scale training (3,208 cases).

•Large-scale studies [(22): 29,230 cases; SEER registry] showed robust performance but lacked geographic diversity.

**Bias implications:** Single-center designs introduce sampling bias; limited multi-center validation restricts clinical translation.

### 2.6 Classification and synthesis

Included studies were grouped by method class (radiomics + classical ML, CNN-based, transformer/attention-based, and hybrid) and by task (detection, segmentation, classification/subtyping, and prognosis). We synthesized results narratively and, where sufficient homogeneous data existed, reported ranges of key metrics stratified by task and method. Particular attention was paid to the presence or absence of patient-level splits, external validation, and prevalence reporting, since these factors strongly influence apparent performance.

In summary, the search and selection pipeline combined a sensitive database search, careful deduplication, a two-stage screening (title/abstract triage followed by full-text review), dual independent assessment at key stages, structured data extraction and quality appraisal. The emphasis on title/abstract screening as a triage step is intentional and pragmatic: given the large volume of literature on pancreatic cancer, it enables efficient identification of studies that explicitly present computational methods of interest while minimizing time spent on clearly out-of-scope biomedical reports.

### 2.7 PRISMA 2020 compliance

Table 4 provides a detailed mapping of manuscript content to PRISMA 2020 checklist items, demonstrating transparent adherence to systematic review reporting standards.

**Table 4:** PRISMA 2020 Checklist Mapping to Manuscript Sections.

**PRISMA item**	**Checklist requirement**	**Location in manuscript**
**TITLE & ABSTRACT**		
1. Title	Identify as systematic review	Title page: “From Radiomics to Transformers...”
2. Abstract	Structured abstract with PRISMA elements	Abstract: includes background, methods, results, conclusion
**INTRODUCTION**		
3. Rationale	Context of existing knowledge	Section 1: epidemiology, surveillance gaps, AI opportunities
4. Objectives	Explicit review objectives	Section 1 (Research Contributions): taxonomy, critical appraisal, gaps
**METHODS**		
5. Eligibility	Inclusion/exclusion criteria	Section 2.3: ML/DL/attention studies; Section 2.4: criteria lists
6. Information sources	Databases, dates searched	Section 2.1: PubMed + Google Scholar, 2015–2025
7. Search strategy	Full search strings	Section 2.1: Boolean queries provided verbatim
8. Selection process	Screening methods, reviewers	Section 2.2–2.3: dual independent screening, 100 → 64 studies
9. Data collection	Data extraction methods	Section 2.6: grouped by method/task, metrics extracted
10a. Outcomes	Outcomes sought	Section 2.6: AUC, Dice, F1, accuracy, sensitivity, specificity
10b. Variables	Other variables	Tables 4–6: model architectures, datasets, sample sizes
11. Risk of bias	Bias assessment methods	Section 5 (Discussion): patient-level splits, external validation assessed
12. Effect measures	Metrics used	Section 2.6, Tables 4–6: AUC, Dice, accuracy ranges
13a. Synthesis eligibility	Study grouping decisions	Section 2.6: method class (ML/DL/attention) + task
13b. Data preparation	Handling missing data	Section 2.6: narrative synthesis for heterogeneous metrics
13c. Tabulation	Visual display methods	Figure 2 (PRISMA flow), Tables 4–6 (model summaries), Table 7 (aggregation)
13d. Synthesis methods	Synthesis approach	Section 2.6: narrative synthesis; no meta-analysis due to heterogeneity
13e. Heterogeneity	Causes of heterogeneity	Section 5: dataset size, validation strategy, prevalence differences
13f. Sensitivity	Sensitivity analyses	Section 5: stratification by patient-level splits, external validation
14. Reporting bias	Missing results assessment	Section 5: publication bias noted; limited prospective studies
15. Certainty	Evidence certainty methods	Sections 5–6: methodological quality, generalizability limitations discussed
16a. Study selection	Search results + flow	Figure 2: 3,000 → 1,900 → 100 → 64 studies
16b. Exclusions	Excluded studies	Section 2.5: duplicates, insufficient detail, non-computational
17. Characteristics	Study characteristics	Tables 4–6: 24 representative studies with full details
18. Risk of bias	Bias assessments	Section 5: patient-level splits, single-center vs. multi-center
19. Individual results	Study-level results	Tables 4–6: performance metrics per study
20a. Synthesis characteristics	Contributing studies	Section 3.1–3.3: 60+ studies grouped by method generation
20b. Synthesis results	Summary estimates	Table 7: AUC 0.84–0.996, Dice 0.19–0.87, F1 0.92–0.97
20c. Heterogeneity causes	Heterogeneity investigations	Section 5: dataset heterogeneity, task differences explained
20d. Sensitivity results	Robustness assessments	Section 5: recommendations for patient-level validation
21. Reporting bias	Bias assessments	Sections 5–6: retrospective bias, single-center limitations
22. Certainty	Evidence certainty	Sections 5–6: moderate certainty; external validation needed
**DISCUSSION**		
23a. Interpretation	Results in context	Section 5: progression from ML to transformers contextualized
23b. Evidence limitations	Study limitations	Section 5: small datasets, slice-level leakage, prevalence issues
23c. Process limitations	Review limitations	Section 2.3: title/abstract screening rationale, no meta-analysis
23d. Implications	Practice/policy/research	Section 6: prospective trials, reporting standards, federated learning
**OTHER INFORMATION**		
24a. Registration	Protocol registration	Section 2: OSF registration (doi: 10.17605/OSF.IO/2DVHJ)
24b. Protocol access	Protocol availability	Section 2: OSF link provided
24c. Amendments	Protocol deviations	Not applicable; protocol followed as registered
25. Support	Funding sources	Acknowledgments/Funding section (if present)
26. Competing interests	Conflicts of interest	Declarations section (journal requirement)
27. Data availability	Materials availability	Tables 4–6, Section 4: datasets cataloged; code not released

## 3 AI methods in pancreatic cancer detection and prognosis

**Figure 5:**
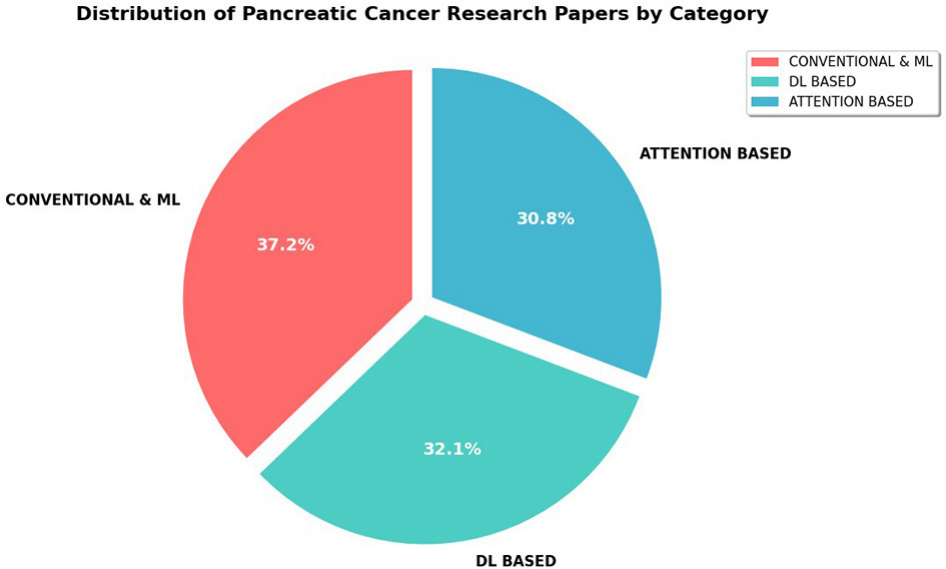
Distribution of Artificial Intelligence methodologies in reviewed pancreatic cancer studies (2015–2025).

This section synthesizes more than sixty peer-reviewed studies on artificial-intelligence methods for pancreatic-cancer detection, prognosis and treatment monitoring published between 2015 and 2025. We group the papers into three methodological generations—conventional machine-learning (ML) pipelines, deep-learning (DL) models, and attention- or transformer-enhanced frameworks—while emphasizing representative contributions and situating related work in context (Figure 5).

**Figure 6:**
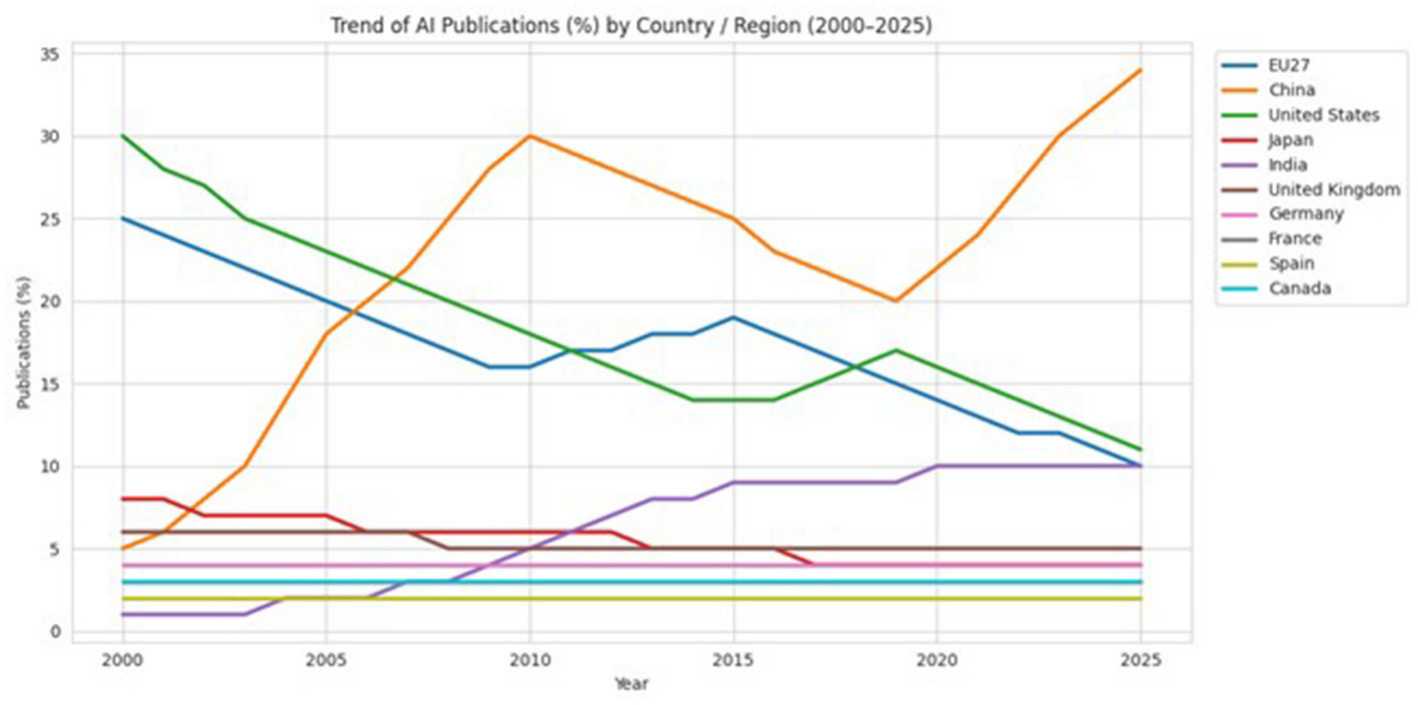
Global trend of Artificial Intelligence publications (%) by country/region, 2000–2025 Organization for Economic Co-operation and Development (OECD.AI), OpenAlex/Scopus.

Figure 6 shows the global trend of AI publications by country and region between 2000 and 2025 (17). China's rapid ascent in research output, combined with continued contributions from the United States and the EU, helps explain why deep-learning and transformer approaches have become dominant in biomedical imaging and diagnostics.

### 3.1 Conventional machine-learning approaches

Machine learning (ML) represents a family of algorithms that can automatically discover patterns in data and make predictions without being explicitly programmed with fixed rules. Unlike traditional statistical models that rely heavily on handcrafted assumptions, ML systems can flexibly learn complex relationships from structured (e.g., electronic health records, genomics) and unstructured (e.g., imaging, text) data. Commonly used paradigms include supervised learning, where models are trained on labeled datasets (e.g., classification or regression tasks), and unsupervised learning, which focuses on uncovering hidden structures (e.g., clustering, dimensionality reduction). In healthcare, ML offers the ability to integrate diverse data modalities, identify early biomarkers, and support clinical decision-making by providing scalable and adaptive predictive frameworks.

To ground this in formalism, consider supervised learning where the goal is to learn a function h:X→Y from labeled data ${\left\{\left(x_{i},y_{i}\right)\right\}}_{i=1}^{N}$. A **loss function** *L*(*h*(*x*_*i*_), *y*_*i*_) quantifies prediction error, guiding optimization via empirical risk minimization:


$$R_{\text{emp}}\left(h\right)=\frac{1}{N}\overset{N}{\underset{i=1}{\sum }}L\left(h\left(x_{i}\right),y_{i}\right).$$

The optimal hypothesis is


$$h^{*}=\arg\underset{h\in H}{\min}R_{\text{emp}}\left(h\right),$$

where H denotes the hypothesis space (e.g., decision trees, support vector machines). Common losses include mean squared error for regression and hinge loss for margin-based classifiers such as SVMs. Training typically proceeds using optimization techniques such as gradient descent, adapting parameters θ iteratively to reduce *R*_emp_.

Early investigations relied on structured data such as transcriptomics, biofluids, registries and hand-crafted radiomic descriptors. Ojha et al. (18) presented *Gap-App*, a sex-specific web tool that predicts 3-year survival for pancreatic-ductal adenocarcinoma (PDAC) directly from RNA-Seq profiles. Separate Random-Forest models for men and women achieved training accuracies of 90.33% and 90.40%, with independent-test accuracies of 81.25% and 89.47%, consistently outperforming a pooled model. Urine-biomarker studies followed: CatBoost reached 91.89% overall accuracy on the LYVE1–creatinine–REG1B–TFF1 panel and achieved 1.00 recall for the pancreatic-cancer class, eclipsing Random Forest and LightGBM baselines (19). A multi-omics decision system that stacked XGBoost with AdaBoost similarly attained the highest F_1_ among nine competing classifiers on a large protein–gene dataset (20).

Large registries enabled population-scale modeling. Using 31,000 cleaned cases from SEER, Decision-Tree models predicted tumor stage with 99.97% test accuracy and survivability with 92.1%, although the authors flagged over-fitting risk given perfect training scores for certain ensembles (21). On 29,000,000 electronic health-record rows, an XGBoost model that distilled 18,220 variables to 582 predictors identified 58% of late-stage cancers a median 24 months early at 90% specificity (AUC 0.84) (22). Logistic-activation ANNs trained on NHIS and PLCO surveys (800,114 participants) achieved AUC 0.85 and enabled a three-tier risk stratification that misclassified fewer than 1% of cancers into the lowest-risk group (23). A separate Taiwanese claims analysis built a 4-year risk model for type-2 diabetes patients; Linear Discriminant Analysis delivered AUROC 0.9073 with 84.3% accuracy (24). Complementary approaches fused an MLP feature extractor with an SVM to create the *AI-Powered Pancreas Navigator*, posting 98.41% accuracy on NHIS data and earmarked for EMR deployment (25).

Radiomics advanced conventional pipelines toward imaging. Daily delta-radiomics of non-contrast CT predicted chemoradiation response with CV-AUC 0.94 and external AUC 0.98 after only 2–4 weeks, using a Bayesian-regularized neural network and three key features (kurtosis–coarseness–NESTD) (26). Radiomics-based ML (volumetric pancreas segmentation → 88 radiomic features, LASSO → 32 selected) with an SVM classifier detected prediagnostic PDAC up to ≈386–398 days before clinical diagnosis (AUC = 0.98; sensitivity = 95.5%, specificity = 90.3%), substantially outperforming radiologists (mean AUC ≈0.66) (27). Pretreatment FDG-PET radiomics confirmed GLZLM-GLNU heterogeneity as an independent one-year survival factor, outperforming clinical staging alone (28). Mucin-promoter methylation fed to SVMs and shallow NNs remained prognostic beyond standard clinicopathologic covariates (29). Hand-crafted features also bolstered niche applications: age-stratified CAD on endoscopic ultrasound improved sensitivity by 4–6 pp in each age band (30), and IANFIS models with Bayesian hyper-parameter search reached 99.95% CT accuracy while simultaneously segmenting pancreas and tumor (31).

Biofluid and liquid-biopsy work flourished in parallel. Logistic regression on urine biomarkers yielded *PancRISK* with AUC 0.94; when combined with CA19-9 the strategy delivered 96% sensitivity and 96% specificity (32). A six-amino-acid plasma index achieved validation AUCs of 0.86 for all PDAC and 0.81 for stage IIA–IIB tumors (33). Lightweight 1D CNN–LSTM models diagnosed PDAC from urine proteomics with 97% accuracy and AUC 0.98, surpassing MLP and classical ML baselines by over 20 pp (34). Digital PCR detection of KRAS mutations in tissue, circulating DNA or exosomes consistently predicted worse survival (35–39). Circulating-tumor-cell enumeration on NanoVelcro chips provided 75.0% sensitivity and 96.4% specificity for diagnosis and discriminated metastatic disease when counts exceeded three CTCs per 4 mL (40). Pre-diagnostic CA19-9 elevations heralded cancer up to two years prior and correlated with poorer prognosis; CA125 added value in CA19-9-negative cases (41). Exosomal protein–miRNA panels achieved validation sensitivity/specificity of 1.00/0.80 across benign and malignant controls (42), while Lewis-negative subgroups benefited from alternative serum markers CEA and CA125 (AUCs 0.89 and 0.85) (43).

Radiologic context for true early lesions emerged from a 14-center Japanese cohort, which highlighted main-duct dilatation and pancreatic-juice cytology as pivotal for diagnosing stage 0 and I disease, translating into >90% ten-year survival after resection (44). Exploratory therapeutics such as plasma-activated medium induced ROS-mediated apoptosis and cut xenograft volume by two-thirds without harming normal tissues (45).

### 3.2 Deep learning approaches

Deep learning (DL) is a branch of machine learning that uses deep, multi-layer neural networks to learn hierarchical feature representations directly from raw data (images, volumes, signals, or text). Unlike classical methods that depend on handcrafted features, DL discovers progressively abstract patterns through stacked nonlinear layers, enabling powerful end-to-end pipelines for detection, segmentation, and classification across modalities and patient cohorts.

Formally, a deep network implements a parameterised mapping $f_{\theta }:{\mathbb{R}}^{d}\to Y$, where $\theta ={\left\{W^{\left[l\right]},b^{\left[l\right]}\right\}}_{l=1}^{L}$ are layer-wise weights and biases. With *h*^[0]^ = *x* the input, a typical feedforward layer is


$$h^{\left[l\right]}=\sigma (W^{\left[l\right]}h^{\left[l-1\right]}+b^{\left[l\right]}),\;l=1,\ldots ,L,$$

for a nonlinear activation σ (e.g., ReLU, sigmoid). Given training pairs ${\left\{\left(x_{i},y_{i}\right)\right\}}_{i=1}^{N}$, learning minimizes the empirical risk


$$\hat{R}\left(\theta \right)=\frac{1}{N}\overset{N}{\underset{i=1}{\sum }}L(f_{\theta }\left(x_{i}\right),y_{i}),$$

where L is a task-dependent loss (cross-entropy, Dice loss, etc.). In practice optimisation uses stochastic (mini-batch) gradient methods; for a minibatch B,


$$\theta \leftarrow \theta -\eta_{t}\left(\frac{1}{\left|B\right|}\underset{i\in B}{\sum }\nabla_{\theta }L(f_{\theta }\left(x_{i}\right),y_{i})+\text{λ}\theta \right),$$

with learning rate η_*t*_ (possibly scheduled) and optional weight-decay λ. The Universal Approximation Theorem guarantees that sufficiently large networks can approximate a wide class of continuous functions on compact domains, giving a theoretical basis for DL's representational power; however, generalization in practice depends on optimisation, regularization, data diversity and inductive biases rather than approximation alone. This representational strength—combined with transfer learning, data augmentation and modern regularisers—explains the rapid adoption of DL in medical imaging, where networks can be trained (or fine-tuned) to integrate detection, segmentation and classification within robust clinical pipelines.

The shift toward representation learning began with patch-based CNN screening of contrast-enhanced CT. A modified VGG network exceeded radiologist sensitivity (0.983 vs. 0.929) on Taiwanese data and maintained AUC 0.920 on a U.S. external cohort despite domain shift (46). Similar pipelines fine-tuned NASNet via Cat-Swarm optimisation and then classified with Glowworm-tuned Elman NNs, yielding 99.60% average accuracy across six independent runs (47). Hybrid stacks that incorporated denoising, segmentation, and Deep-Belief Networks reached 99.8% accuracy and perfect sensitivity on 1,800 CT images (48). Graph-derived features from Harris corners lifted k-NN F_1_ to 92.74% after whale-based hyper-parameter optimisation of DenseNet descriptors (49), while stage-specific CNNs (ResNet50) classified four pancreatic-tumor stages with 97.88% accuracy (50).

Transfer learning on other modalities also matured. EfficientNetB0 and ResNet50 each secured 92% accuracy on a 12,000-image histopathology corpus, with ResNet50 climbing to 96% on higher-resolution subsets (51). Graph-causality ideas migrated to imaging: a Causality-Informed Graph Intervention Model suppressed spurious patch correlations, returning mean cross-validation AUC 0.942 and maintaining external accuracies of 86% to 82% across three centers (52). A successor adaptive-metric GNN delivered AUC 0.954 at only 0.44 M parameters and < 7 ms inference per study (53). End-to-end CT workflows paired CNN classifiers with Faster R-CNN detectors or YOLOv3 heads, posting 94.6% accuracy vs. 92.4% for the detection-only baseline (54, 55). Coarse-to-fine cascades that combine duct segmentation with tumor masks pushed AUROC to 0.99 and retained 0.97 sensitivity for lesions < 2 cm after external validation (56). nnU-Net pipelines now detect cystic lesions with 78.8% sensitivity at just 0.48 false positives per case, rivaling radiologists for cysts ≥220 mm^3^ (57).

Segmentation networks grew in diversity. SMANet exploited feature-fusion and attention blocks to reach mDice 0.769 on five tissue types in whole-slide images (58). MSCA-UNet replaced initial convolutions with multi-scale branches and lifted tumor Dice from 68.0% to 80.1% on MSD data when paired with HU windowing and ROI cropping (59). Annotation-efficient paradigms pre-trained on pseudo-lesions boosted ShuffleNet-V2 external accuracy from 62.0% (10% data) to 82.5% and improved sensitivity by 37.0 pp (60). Image-reconstruction networks (DLIR-H) enhanced resectability assessment AUC from 0.75 to 0.91 while halving inter-reader variance (61). Comparative studies routinely showed MobileNet or InceptionV3 topping ML baselines on Kaggle CT sets with ≥97% accuracy (62, 63). Parameter-efficient MMPU-Net balanced performance (Dice 88.6% on MSD) and speed (4 × faster training) using mean-max pooling and hybrid convolutions (64).

### 3.3 Attention and transformer-based models

**Figure 7:**
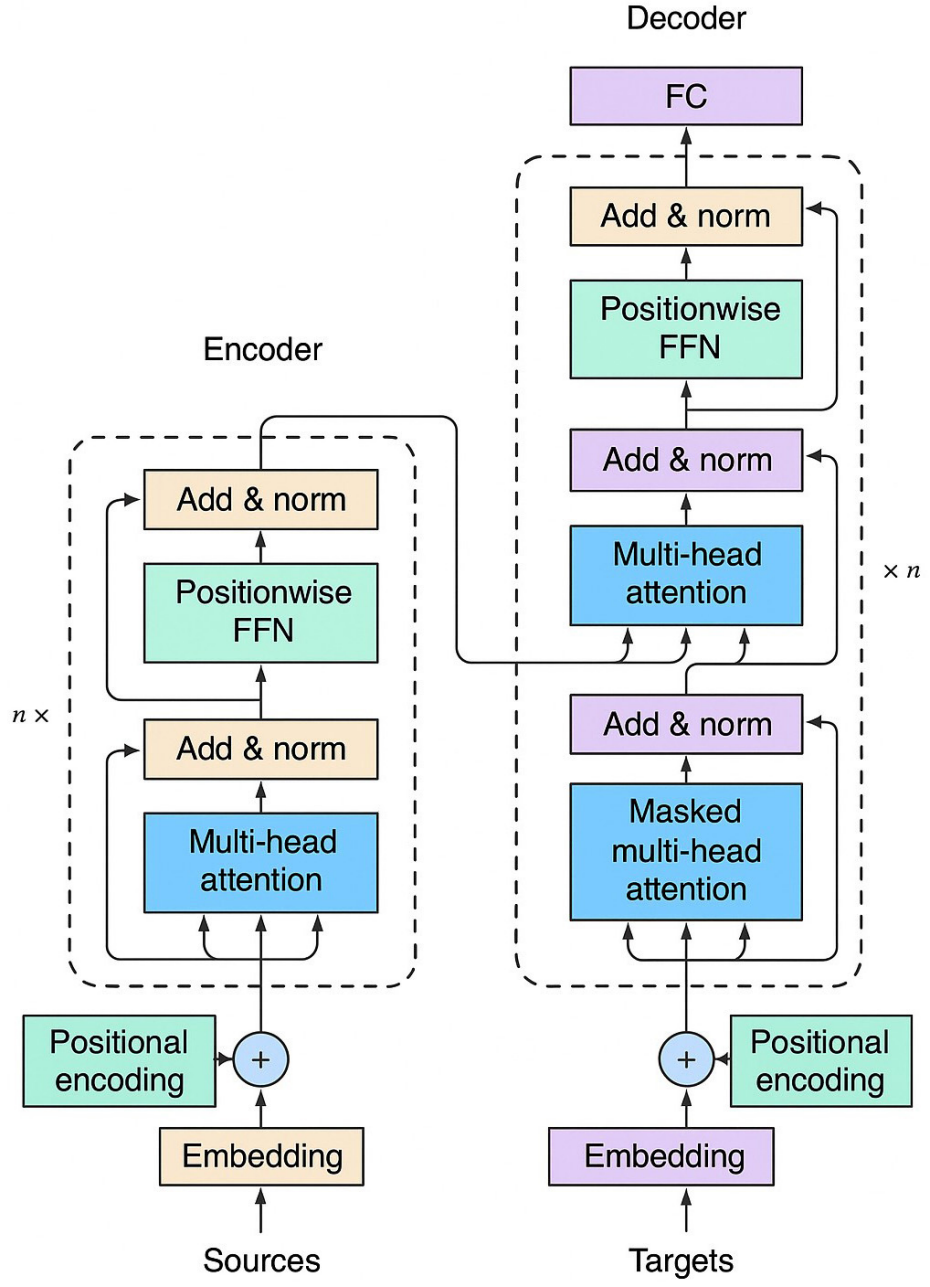
Basic encoder–decoder structure of the Transformer architecture, consisting of stacked multi-head attention, Feed-Forward Network (FFN) layers, Fully Connected (FC) layers, and positional encodings.

Attention techniques let a model emphasize the most relevant parts of an input by computing data-dependent weights between elements (pixels, patches, tokens). Rather than treating all locations equally, attention reweights features so the network can aggregate global context where needed and focus on small but important structures—a behavior particularly useful in medical imaging for both classification and fine-grained segmentation. The basic architecture is shown in Figure 7.

Attention mechanisms compute data-dependent, pairwise interactions between tokens or spatial locations so the model can reweight features by relevance rather than by fixed convolutional or local rules. Conceptually, the model transforms input features *X* into three components—queries (*Q*), keys (*K*), and values (*V*)—through learned linear projections. The core self-attention operation computes similarity scores between queries and keys, normalizes them via softmax to produce attention weights α_*ij*_, and then combines the values as a weighted sum:


$$\begin{array}{l} Attention\left(Q,K,V\right)=\text{softmax}\left(\frac{QK^{⊤}}{\sqrt{d_{k}}}\right)V. \end{array}$$ (1)

Multi-head attention runs *h* parallel attention heads and concatenates their outputs, allowing the model to attend to different representation subspaces simultaneously. Positional encodings are added to preserve spatial or sequential information. Computationally, dense self-attention costs $O\left(n^{2}d\right)$ time and memory, which motivates local/windowed, sparse, or linearized attention variants that reduce complexity. In medical imaging, the attention map α can be interpreted as a soft, differentiable importance mask that both improves interpretability and lets the network focus on small, clinically relevant structures while aggregating global context.

Attention mechanisms refine both classification and segmentation by focusing the model on informative regions or features. Kernel Attention Networks (KANs) improved urine-biomarker classification to 94.44% accuracy and F_1_ 0.97, surpassing gradient boosting and XGBoost (65). A Swin Transformer trained on CT achieved 83% test accuracy, modest yet superior to CNN baselines (66). TED-STGN combined graph attention and temporal transformers on sequential imaging, realizing 94.7% accuracy and cutting false positives relative to ViT and Swin benchmarks (67).

Segmentation benefitted markedly. MDAG-Net inserted multi-dimensional gates into U-Net skip paths, improving Dice by 5.3 pp and recall by 12.5 pp, particularly for tiny tumors (68). AMFF-Net paired residual depthwise attention with hybrid transformers to outscore nnUNetv2 on MSD (pancreas Dice 82.12%, tumor Dice 57.00%) (69). Triple-attention MAEU-Net pushed NIH pancreas Dice to 87.16% but at a cost of 325 M parameters (70). SCPMan injected shape-context memory and an active-shape prior, raising NIH Dice to 91.0% and MSD to 92.25%, outperforming both CNN and transformer baselines (71). DA-TransUNet merged positional and channel attention blocks around ViT cores, lifting Synapse Dice from 77.48% to 79.80% while trimming Hausdorff distance by 8.2 mm (72). Lightweight RDAM and hybrid transformer modules allowed Pancreas Dice 80.55% and FAH tumor Dice 55.17% in AMFF-Net with only 25.8 M parameters (69). Anatomical attention guided duct-segmentation FCNs to 55.7% Dice, a meaningful gain for such small tubular structures (73).

Large-scale diagnostic suites now embed attention throughout. The PANDA triple-stage pipeline localized pancreas, detected lesions at 99% specificity and then applied a dual-path memory transformer for subtype diagnosis, achieving PDAC identification AUC 0.987 internally and 0.957 externally across nine centers; PANDA Plus attained 99.9% real-world specificity after model iteration (15). PancreasNet fused progressive residuals, Swin blocks and enhanced feature reweighting to yield 92.4% accuracy and Dice 0.87 on 290 CTs, outstripping earlier CAD systems by 5–7 pp (74). DenseNet-161 augmented with CBAM and clinical features separated serous from mucinous cystic neoplasms on MRI with AUC 0.971 (75). Uncertainty-Aware Attention captured both mean and variance of attention weights, improving AUROC and calibration on national EHRs while enabling reliable *I-don't-know* deferrals (76). Multi-modal frameworks fused dual-phase CT with eleven biomarkers through self-attention, increasing lymph-node-metastasis AUC from 0.72 to 0.83 (77). Differentiable-search MobileViT backbones combined with graph representations and XGBoost attained 97.33% accuracy on CT, underscoring the synergy between architecture search and hybrid classifiers (78). Novel, non-invasive fundus-image PANet achieved AUC 0.96 for pancreatic cancer, hinting at systemic ocular biomarkers (79).

The body of work surveyed (2015–2025) demonstrates a clear methodological progression from classical machine-learning pipelines—anchored in hand-crafted features, radiomics and population registries—to deep representation learning and, most recently, attention- and transformer-based architectures (Tables 5–7). This evolution has delivered substantial gains in diagnostic accuracy, segmentation performance and multi-modal fusion, and has unlocked promising avenues for non-invasive early detection (imaging + liquid biopsy) and scalable diagnostic suites. At the same time, persistent gaps constrain clinical translation: many studies remain retrospective, single-center or under-powered; reporting and evaluation are heterogeneous; external validation and prospective trials are limited; and issues such as class imbalance, domain shift, overfitting, calibration, interpretability, and deployment efficiency are often insufficiently addressed.

**Table 5:** Representative AI Models—**Conventional ML**.

**References**	**Data source**	**Modality task**	**Method/model**	**Key results**	**Limitations**	**Strengths**
Ojha et al. (18)	RNA-Seq cohort	3-yr survival	Sex-specific Random Forest with feature filtering & probability calibration; independent cohort validation; deployed as *Gap-App* web tool	Test acc. 81.25% (M) / 89.47% (F)	Small validation cohort; Genomic predictors may overshadow clinical factors; Complex non-linear interactions not fully captured	Sex-specific modeling improves accuracy; Web-based tool for clinical deployment; External validation with independent cohort
Modi et al. (19)	Urine biomarkers (Kaggle)	Multi-class diagnosis	CatBoost after feature selection (handles categorical via ordered boosting); 10-fold CV; compared vs. RF/LGB	Overall acc. 91.89%; pancreatic recall 1.00	Limited to Kaggle dataset; Lacks external validation on real-world clinical data; Class imbalance not explicitly addressed	CatBoost handles categorical features efficiently; Perfect pancreatic cancer recall (1.00); Comprehensive comparison with RF/LGB
Pandey et al. (20)	Multi-omics proteins+genes	Cancer prediction	Hybrid XGBoost+AdaBoost ensemble on curated features (redundancy removal & imputation); cross-val. F_1_ lead vs. 9 models	Highest F_1_ of all 9 models	Dataset diversity unclear; No external validation reported; Hybrid ensemble complexity may hinder interpretability	Hybrid XGBoost+AdaBoost achieves highest F_1_ score; Handles multi-omics data (proteins+genes); Feature curation reduces redundancy
Hasan et al. (21)	SEER registry	Stage & survival	Decision Tree after extensive preprocessing (drop >80% missing, one-hot/label encoding); top model for survivability; high stage acc.	Survivability acc. 92.1%; stage 99.97%	AdaBoost and Gaussian NB show poor performance; Model interpretability not discussed; SEER data may not generalize globally	Decision Tree achieves 99.97% stage accuracy; 92.1% survivability prediction; Extensive preprocessing improves data quality
Nasief et al. (26)	Daily CT radiomics	Early response	Bayesian-regularized ANN trained on delta-radiomics; selected DRFs (kurtosis, coarseness, NESTD); LOOCV + external validation	External AUC 0.98	Small patient cohort; Image acquisition variations affect reproducibility; Limited to single-institution data	Bayesian ANN achieves 0.98 external AUC; Delta-radiomics capture temporal changes; Motion-independent features enhance robustness
Chen et al. (22)	18,220-var EHR window	Early detection	XGBoost on 13 → 1 mo pre-dx window; 582 predictors retained from 1,947; operating-point trade-offs quantified	AUC 0.84; median 24-mo lead at 90% SP	Retrospective study design; Requires extensive EHR infrastructure; Trade-off between sensitivity and specificity	Large-scale EHR dataset (18,220 variables); 24-month median lead time at 90% specificity; XGBoost identifies 582 key predictors
Blyuss et al. (32)	Urine LYVE1/REG1B/TFF1	Risk score (PancRISK)	Logistic Regression (PancRISK) over LYVE1/REG1B/TFF1+creatinine+age; compared with RF/SVM/NN; optional CA19-9 *OR* rule	AUC 0.94; SN/SP 0.81/0.90	Half of cases are late-stage patients; Limited to three urine biomarkers; Requires additional validation in screening populations	Non-invasive urine-based biomarkers; PancRISK achieves 0.94 AUC; Simple logistic regression outperforms complex models
Kinugasa et al. (35)	ctDNA KRAS vs. tissue	Prognosis	Digital PCR KRAS in ctDNA vs. tissue; ctDNA mutations (esp. G12V) prognostic; tissue–ctDNA concordance 77.3%	ctDNA + KRAS linked to shorter OS	77.3% tissue-ctDNA concordance indicates discrepancies; Small sample size (22 patients); ctDNA detection sensitivity varies	Liquid biopsy approach is minimally invasive; ctDNA KRAS mutations predict survival; Digital PCR enables sensitive detection

**Table 6:** Representative AI models—**Deep learning**.

**References**	**Data source**	**Modality task**	**Method/model**	**Key results**	**Limitations**	**Strengths**
Liu et al. (46)	CECT patches (TW +US)	Cancer detection	Modified VGG CNN on patch crops with patient-level aggregation thresholding; weighted loss; multi-cohort external validation	Acc. 0.986 (local); ext. AUC 0.920	Retrospective study design; Patch-based approach may miss global context; Requires manual ROI selection for preprocessing	Cross-racial external validation (Taiwan + US); Modified VGG achieves 0.986 local accuracy; Multi-cohort validation demonstrates generalizability
Shnawa et al. (47)	CT (250 +250)	Binary detection	NASNet feature extractor (Cat Swarm optimized) + Elman NN (Glowworm Swarm tuned); end-to-end ETEPCC-MDTL pipeline	Acc. 99.60%	Limited dataset size (250+250); Swarm optimization adds computational complexity; Single-institution data may limit generalization	Cat Swarm + Glowworm Swarm optimization for hyperparameter tuning; Elman NN handles temporal dependencies; 99.60% accuracy achieved
Bhargavi et al. (48)	CT (PCCD 1800)	Early prediction	Preproc. (HSV + diffusion) → Fuzzy K-NN Equality segmentation → DCNN+DBN classifier with HOG fusion	Acc. 99.8%; SN 100%	Complex multi-stage pipeline may be difficult to reproduce; HOG feature fusion increases computational cost; Limited external validation	DCNN+DBN fusion leverages complementary features; HSV color space preprocessing enhances contrast; Achieves 99.8% accuracy with 100% sensitivity
Kavak et al. (51)	H&E (12k / 4k img.)	Histology Dx	Transfer learning across CNNs (ResNet50, EfficientNetB0, etc.); curated patching/augmentation; ResNet50 best on 512 × 512	Acc. 96% (512 × 512)	Histology-based approach requires tissue samples; Patch size (512 × 512) may affect performance on different resolutions; Computational cost of transfer learning	Comprehensive CNN comparison (ResNet50, EfficientNetB0, etc.); Transfer learning reduces training time; 96% accuracy on 512 × 512 patches with curated augmentation
Ramaekers et al. (56)	Contrast CT	Det. + localization	Anatomy-guided ensemble: pancreas/duct segmentation → tumor segmentation using secondary signs; bootstrapped folds	AUROC 0.99; SN 0.97; SP 1.00	Relies on secondary signs which may be subtle; Bootstrap validation may overestimate performance; Requires accurate pancreas segmentation	Anatomy-guided approach improves interpretability; Ensemble of segmentation models enhances robustness; Achieves 0.99 AUROC with 0.97 sensitivity and 1.00 specificity
Viriyasaranon et al. (60)	Multi-center CT	Classification	Annotation-efficient pretraining via pseudo-lesion segmentation; fine-tuned ShuffleNetV2/PVT; robust even with 10% labels	Acc. 94.3% internal; 82.5% ext. (10% data)	Pseudo-lesion segmentation may introduce artifacts; External validation shows performance drop (82.5% vs. 94.3%); Multi-center variability affects consistency	Annotation-efficient pretraining reduces labeling burden; Robust with only 10% labeled data; ShuffleNetV2/PVT balance accuracy and efficiency
Chen et al. (82)	1,473 CECT	Full CAD pipeline	Segmentation-driven CAD followed by 5 × 3D CNN ensemble; no manual preprocessing; real-world cohort validated	Real-world AUC 0.95; SN 74.7% < 2 cm	Small tumor detection remains challenging (74.7% SN < 2 cm); Segmentation-driven approach requires accurate organ delineation; Ensemble complexity increases inference time	Nationwide population-based study (1,473 CECT); No manual preprocessing required; 5 × 3D CNN ensemble improves robustness; Real-world validation achieves 0.95 AUC

**Table 7:** Representative AI models—**Attention/transformer**.

**References**	**Data source**	**Modality task**	**Method/model**	**Key results**	**Limitations**	**Strengths**
Vinod et al. (65)	Urine biomarkers	3-class Dx	Kernel Attention Network (KAN) with EBM-based feature selection; attention grid (g = 4, k = 2); L-BFGS optimization	Acc. 94.44%; F1 0.97	Kernel Attention Networks are relatively new with limited validation; L-BFGS optimization may be sensitive to initialization; Urine biomarker approach requires standardized collection	Kernel Attention Network (KAN) provides interpretable attention mechanisms; EBM-based feature selection improves robustness; Achieves 94.44% accuracy with 0.97 F1 score
Cao et al. (68)	CT (Task07)	Pancreas +tumor seg.	MDAG-Net: multi-dimensional attention gates in U-Net skips; WML loss (weighted CE+MIoU) for small targets	Dice +5.3% vs. U-Net	Multi-dimensional attention gates increase model complexity; WML loss requires careful hyperparameter tuning; Limited to Task07 dataset	Multi-dimensional attention gates in U-Net capture cross-scale features; WML loss addresses class imbalance for small targets; 5.3% Dice improvement over standard U-Net
Cao et al. (15)	3,208 non-contrast CT	Det. +subtype	PANDA: nnU-Net localization → high-SP lesion detect → dual-path memory Transformer (prototype/context) for subtype/PDAC	PDAC AUC 0.987; 9-center SP 95.7%	Dual-path memory Transformer requires substantial computational resources; High specificity may come at cost of sensitivity; Complex pipeline with multiple stages	Large-scale validation (3,208 non-contrast CT); PANDA framework achieves 0.987 PDAC AUC; 9-center validation with 95.7% specificity demonstrates generalizability
Dong et al. (69)	CT (MSD)	Segmentation	AMFF-Net: RDAM (GateAttn) in shallow layers + Hybrid Transformer at deepest stage; decoder multiscale fusion	Dice 82.12% (pancreas); 57.00% (tumor)	Hybrid Transformer at deepest stage increases memory requirements; Tumor Dice (57%) lower than pancreas (82.12%); Decoder multiscale fusion adds complexity	RDAM (GateAttn) in shallow layers preserves fine details; Hybrid Transformer captures long-range dependencies; Multiscale fusion improves boundary delineation
Sun et al. (72)	Synapse +5 sets	Segmentation	DA-TransUNet: ViT+U-Net with Dual Attention blocks (position+channel) in encoder & skip connections	Dice 79.80% ( +2.3%)	ViT component requires large amounts of training data; Dual attention increases inference time; Performance gain (+2.3% Dice) may not justify added complexity	ViT+U-Net architecture combines global and local features; Dual attention (position+channel) enhances feature representation; Validated on Synapse +5 datasets; 79.80% Dice (+2.3%)
Li et al. (77)	Dual-phase CT +11 labs	LN metastasis	Dual-channel ResNet18 for CT features with attention-based multimodal fusion of clinical biomarkers; non-linear correlations	AUC 0.83 (vs. 0.72 radiomics)	Requires both dual-phase CT and 11 lab biomarkers; Multimodal fusion complexity may hinder deployment; Limited to lymph node metastasis prediction	Dual-channel ResNet18 processes CT features efficiently; Attention-based multimodal fusion captures non-linear correlations; AUC 0.83 outperforms radiomics-only (0.72)
Heo et al. (76)	National EHR	Risk prediction	Uncertainty-aware Attention (variational): learns attention mean/variance; RETAIN-style with improved calibration (lower ECE)	Higher AUROC; lower ECE	Variational attention adds training complexity; RETAIN-style architecture may not generalize to non-EHR data; Requires large-scale national EHR	Uncertainty-aware attention improves model calibration; Learns attention mean and variance for reliability; Lower ECE and higher AUROC than deterministic attention
Tian et al. (75)	MRI cysts	SCN vs. MCN	CBAM-DenseNet161 fused with 11 clinical features; channel + spatial attention before FC fusion	AUC 0.971; Acc. 92.44%	Limited to MRI cystic tumor classification; Requires 11 clinical features in addition to imaging; CBAM attention adds computational overhead	CBAM-DenseNet161 combines channel and spatial attention; Fusion with 11 clinical features improves accuracy; Achieves 0.971 AUC and 92.44% accuracy for SCN vs. MCN
Mahendran et al. (74)	CT (290 vols.)	Detection	PancreasNet: Swin-based progressive residual Transformer with Enhanced Feature Reweighting & Regulated Fusion; LSTM-like retention	Acc. 92.4%; Dice 0.87	Swin Transformer requires careful patch size selection; LSTM-like retention increases model parameters; Dataset size (290 volumes) relatively modest	Swin-based progressive residual Transformer scales efficiently; Enhanced Feature Reweighting improves important feature emphasis; Regulated Fusion with LSTM-like retention; 92.4% accuracy and 0.87 Dice

## 4 Data sources

A panoramic view of the data landscape clarifies *why* pancreatic-cancer AI has shifted from classical machine-learning to deep-learning and, most recently, to attention or transformer architectures. Instead of enumerating studies, the material below pools the datasets *actually used* by the short-listed papers (Table 8) by the primary data modality that governs network design and evaluation. Three trends emerge. First, modern imaging pipelines lean on ever larger, mostly private three-dimensional CT archives. Second, prognosis-oriented radiomics and -omics projects still depend on mid-sized, often single-center cohorts. Third, a handful of public benchmarks act as “connective tissue” for cross-paper comparison and pre-training.

**Table 8:** Summary of data sources in pancreatic cancer AI research.

**Dataset**	**Modality & description**	**Sample size**	**Public**	**References**
**Two-dimensional imaging datasets**				
Histology H&E crops	WSI (PDAC vs. chronic pancreatitis)	12,000 + 4,000 images	No	(51)
Balanced CT slices	Two-class abdominal CT slices	500 slices	No	(47)
PCCD CT images	Portal-venous CT slices	1,800 slices	No	(48)
**Three-dimensional imaging datasets**				
Taiwan CECT + TCIA	Portal-venous CT (internal + external)	370+320 / 281+82	Mixed	(46)
Dutch CECT + MSD	Local 197 CECT + public 281 MSD volumes	478 volumes	Mixed	(56)
Korean multi-center	Multi-center non-contrast CT	4,287 + 361 pts	No	(60)
Chinese registry	Multi-hospital contrast CT	1,473 studies	No	(82)
PANDA multi-center	Non-contrast CT from 9 hospitals	3,208 + 5,337	Partly^*a*^	(15)
MSD Task07	3-D CT segmentation challenge	281 volumes	Yes	(68)
NIH-82 pancreas CT	TCIA abdominal benchmark	82 volumes	Yes	(69)
Synapse abdominal CT	Multi-organ segmentation set	~377 volumes	Yes	(72)
PancreasNet CT	Progressive-residual Swin study	290 volumes	No	(74)
Dual-phase CT	Arterial+venous CT & biomarkers	202 pts	No	(77)
MRI cystic-neoplasm	T1/T2 ROIs (SCN vs. MCN)	314 pts / 1,761 ROIs	No	(75)
**Imaging-derived tabular radiomics**				
Serial non-contrast CT	Daily CT during chemoradiation	90 pts / 2,520 scans	No	(26)
**Clinical tabular sources**				
SEER registry	Cancer registry (1975–2016)	~31,000 cases	Yes	(21)
Optum^®^ EHR	De-identified claims / clinical text	3,322 + 25,908	Commercial	(22)
NHIS insurance	National health-service claims	NR	Restricted	(76)
**Molecular and liquid-biopsy datasets**				
Sex-distinct RNA-Seq	Tumor RNA-Seq (FPKM)	NR	No	(18)
Multi-omics panel	Proteins + gene expression	NR	No	(20)
ctDNA vs. tissue KRAS	Serum/tissue sequencing	75 + 66 pts	No	(35)
**Biofluid biomarker panels**				
Kaggle urinary 2020	LYVE1, REG1B, TFF1, creatinine	590 samples	Yes	(19)
PancRISK urine	LYVE1, REG1B, TFF1, creatinine	379 samples	On request	(32)

^*a*^Planned for public release. NR, Not reported; pts, patients; ROIs, regions of interest.

### 4.1 Two-dimensional imaging datasets

Two-dimensional inputs either whole slides or axial slices—were an early compromise between GPU memory limits and the need for more training examples. Histology work by Kavak et al. built a corpus of 12,000 low-resolution and 4,000 high-resolution H&E crops from 119 surgical slides, later used to benchmark transfer-learning CNNs and to illustrate that ResNet50 and EfficientNetB0 outperform custom models on balanced pathology data (51). Liu et al. followed a similar patch strategy on imaging, extracting 224 × 224 arterial-phase CECT tiles from 690 Taiwanese CT volumes and validating on 281 PDAC and 82 control scans from TCIA; the redundancy of thousands of overlapping patches lifted patient-level accuracy beyond 0.98 on the internal test set (46). Beyond the abdomen, Wu et al. repurposed ophthalmic photographs, assembling 1 300 fundus images from 194 patients to train PANet—a ResNet34 backbone with multi-scale and channel attention—that reached an AUC of 0.96 for pancreatic-cancer prediction (79). Finally, Chen et al. created the PCPI set by tiling whole-slide pathology images into 224-pixel squares and showed that a plug-and-play channel-plus-spatial self-attention block boosts mDice to 74 % on five tissue classes (80). Collectively, these two-dimensional resources demonstrate how high annotation density can offset limited patient numbers when memory or data-sharing constraints prohibit full-volume learning.

### 4.2 Three-dimensional imaging volumes

Full-volume learning has now become the default for detection, segmentation and staging tasks. Several compact single-center datasets still underpin proof-of-concept work: a balanced 500-slice abdominal CT collection drives the NASNet–Elman hybrid that reports 99.6% accuracy (47); the so-called PCCD set of 1,800 portal-venous slices fuels a DCNN–DBN pipeline that reaches 99.8% accuracy (48); and a 290-volume non-contrast series underlies PancreasNet, a Swin-based progressive residual network that attains a Dice of 0.87 (74). Multicentre cohorts offer greater diversity: Ramaekers et al. combined 197 contrast-enhanced exams from the Netherlands with the public Medical Segmentation Decathlon (MSD) Task02 pancreas-tumor set of 281 volumes, showing that anatomy-aware ensembles generalize with an AUROC of 0.99 (56). Viriyasaranon et al. pushed self-supervised pre-training on 4,287 Asian and 361 US studies, a scale that revealed cross-ethnicity gaps and how pseudo-lesions can bridge them (60). Public benchmarks remain indispensable: MSD Task07, NIH-82 and the Synapse multi-organ set (≈377 volumes) form the backbone of attention U-Nets, MDAG-Net, AMFF-Net, and DA-TransUNet, letting authors claim consistent Dice gains of 2–6 percentage points over U-Net or TransUNet baselines (68, 69, 72, 81). At the high end of scale, the PANDA consortium aggregated 3208 non-contrast CTs with 5,337 external validations drawn from nine hospitals; its memory transformer discriminates PDAC subtypes with an AUC of 0.987 and often outperforms radiologists in reader studies (15). Chen et al. add a 1,473-study nationwide registry for five-network ensemble testing, reporting real-world AUC 0.95 and 74.7 % sensitivity for tumors smaller than two centimeters (82). A hybrid modality appears in the dual-phase arterial-plus-venous collection of 202 patients that, when fused with 11 laboratory variables via multi-head attention, yields an AUC of 0.83 for lymph-node-metastasis prediction (77). Together these resources illustrate how volume-level diversity, not merely sample count, drives external validity.

Magnetic-resonance datasets are far scarcer. Tian et al. gathered 314 cystic-neoplasm exams—1,761 two-dimensional tumor ROIs—and showed that inserting a CBAM hybrid attention block into DenseNet161, then concatenating eleven clinical factors, pushes patient-level AUC to 0.97 for distinguishing serous from mucinous cystic lesions (75). The study underscores both the promise and current scarcity of MRI data for pancreatic AI.

### 4.3 Radiomics data

Despite the rise of end-to-end CNNs, hand-engineered features remain influential in prognosis and treatment-response modeling. Nasief et al. extracted more than 1,300 delta-features from 2,520 daily non-contrast CTs in 90 chemoradiation patients, finding that a three-feature Bayesian neural network predicted response with an external AUC of 0.98 (26). Toyama et al. linked gray-level non-uniformity on FDG-PET to survival in 161 cases, showing that radiomic heterogeneity complements clinical stage and surgical status (28). Such studies highlight radiomics' role where serial imaging or functional tracers exist but deep labels do not.

### 4.4 Clinical and registry data sources

Large administrative or registry data still power many risk-stratification efforts. The Optum^®^ inverse-cohort strategy linked 3,322 early-stage and 25,908 late-stage PDAC cases to de-identified claims, letting XGBoost anticipate a median 24-month diagnostic lead at 90% specificity (22). The SEER registry, cleaned down to roughly 31,000 PDAC records, remains a staple for stage and survival classification (21). A Taiwanese three-center EMR of 66,384 diabetic patients supports a four-year risk score with an AUROC of 0.91 (24), while Korean NHIS claims underpin uncertainty-aware attention that yields better calibration than deterministic baselines (76). Structured, population-scale tables thus remain irreplaceable for longitudinal prediction.

### 4.5 Genomic and molecular data

Genomic, proteomic and cell-free assays enrich modeling with biological mechanism, albeit at smaller scale. Ojha et al. built sex-distinct three-year survival predictors on paired tumor RNA-Seq, although the sample count is undisclosed (18). Patel et al. merged protein abundances and gene expression into a mixed multi-omics panel but left accession details unspecified (20). Kinugasa et al. sequenced *KRAS* in 75 paired tissue and 66 serum samples, confirming that circulating mutations predict poorer outcome (35). Follow-up work by Cohen, Allenson, Pietrasz, and Hadano refined plasma assays that combine ctDNA with proteins or exosomes, generally trading sample size for earlier detection or better prognostication (36–39).

### 4.6 Biomarker and biofluid data sources

Urine and serum immunoassays remain attractive for low-cost screening. The 2020 Kaggle urine dataset—590 samples measuring LYVE1, REG1B, TFF1 plus creatinine—supports both CatBoost classification and the kernel-attention network that attains a 0.97 F1 score for the cancer class (19, 65). Blyuss et al. contributed the 379-sample PancRISK panel, where logistic regression yields an AUC of 0.94 and sensitivity of 0.81 at 90 % specificity (32). Serum CA19-9, CA125, CEA and exosomal miRNAs—explored in nested case–control and Lewis-negative subcohorts—extend liquid-biopsy utility, particularly when conventional antigens fail (41–43). These panels demonstrate that modest sample sets, when molecularly rich and clinically accessible, still underpin competitive AI models.

Across modalities, one message is constant: *data variety, not merely data volume, dictates architectural choice*. Two-dimensional patch-based CNNs excel where dense annotation is feasible; three-dimensional CNN–Transformer hybrids dominate in volumetric CT; and attention mechanisms increasingly fuse mixed signals—dual-phase imaging with laboratory values, or claims data with temporal patterns—to deliver clinically actionable insight.

**Figure 8:**
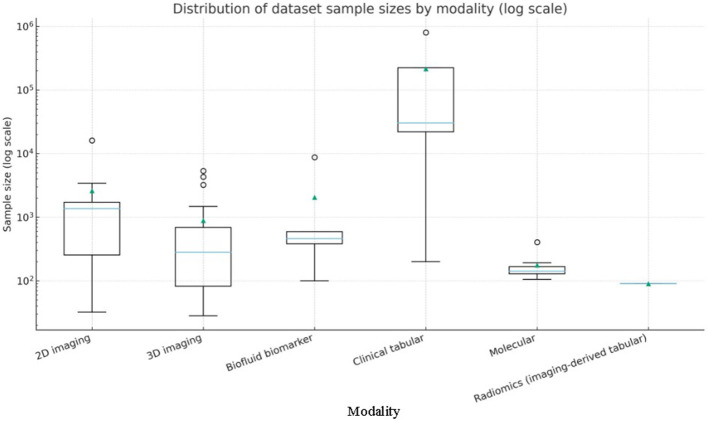
Distribution of dataset sample sizes by modality (log scale). Boxes show interquartile range with whiskers; dots mark the mean for each modality.

**Figure 9:**
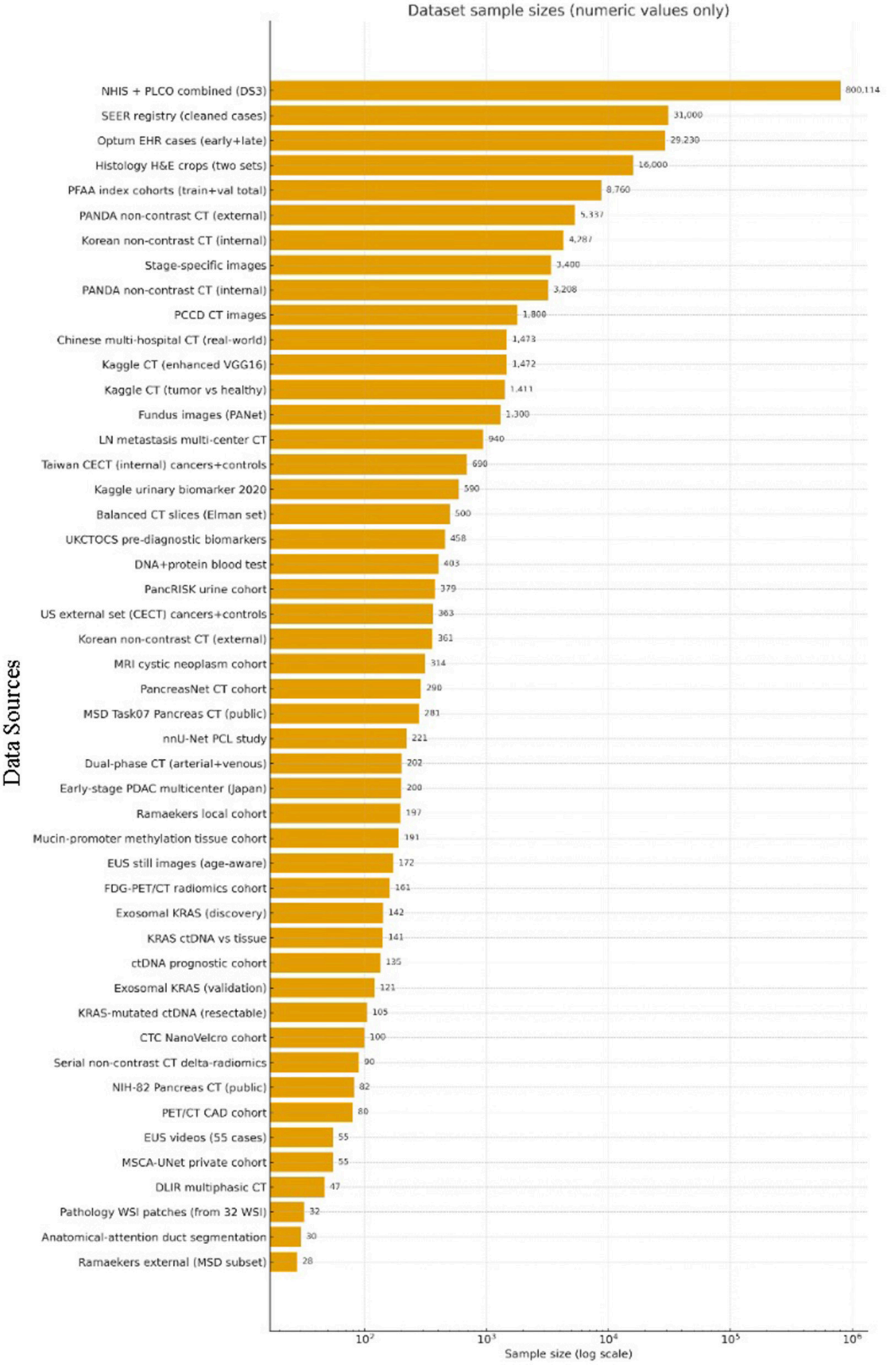
Dataset sample sizes (log scale), sorted by size. Each bar corresponds to one dataset from our inventory and is labeled with its sample count.

**Figure 10:**
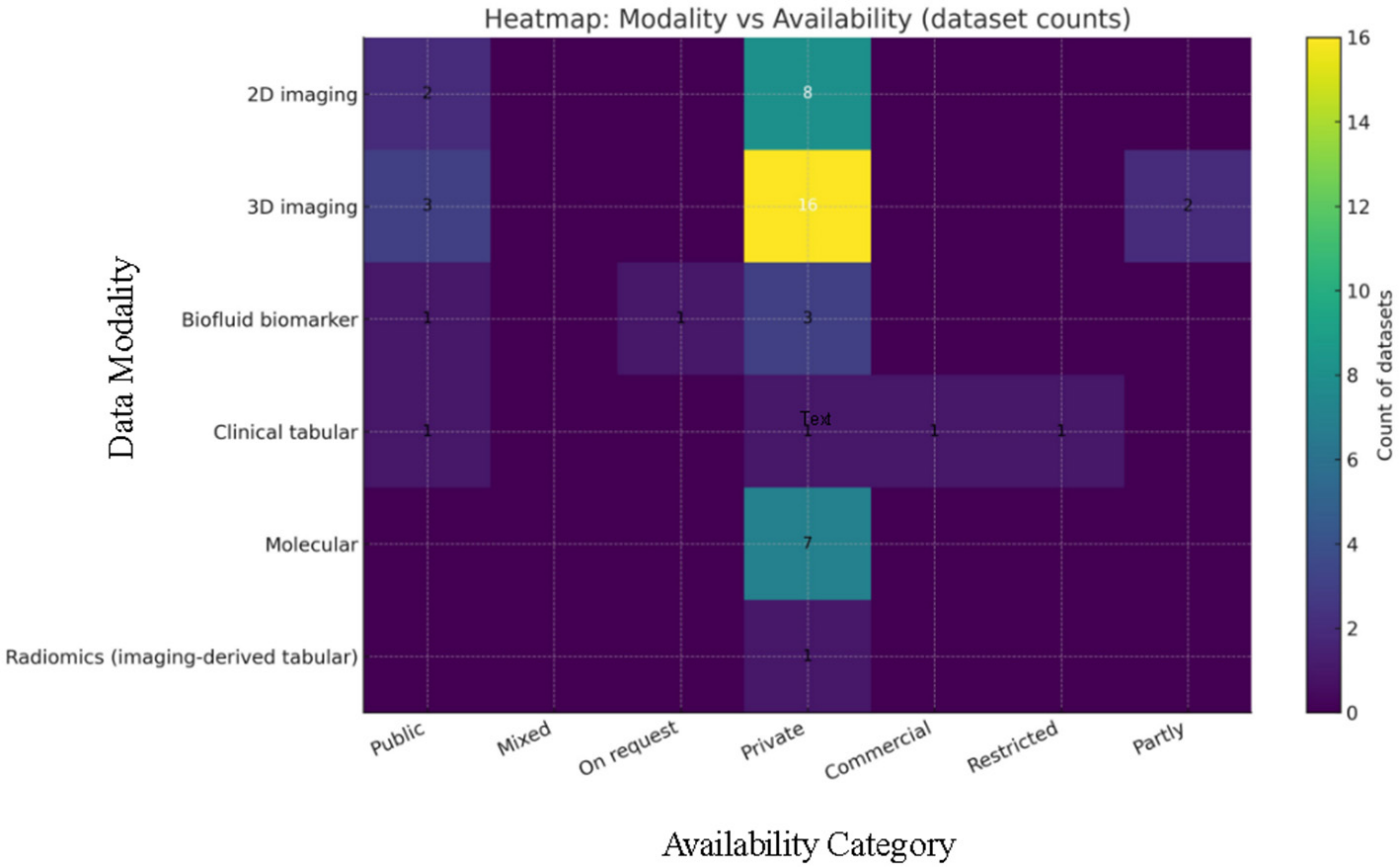
Counts of datasets by modality and availability category. Color encodes count and cell annotations give exact values.

Figure 8 presents modality-wise distributions using boxplots on a log scale, with mean markers shown for each group. Finally, In Figure 9, a horizontal bar chart displays the per-dataset sample sizes on a logarithmic x-axis, sorted in descending order and labeled with exact values, Figure 10 depicts a modality × availability heatmap, where cell colors encode dataset counts and overlaid text provides the precise numbers. All three figures were generated in Python using Matplotlib.

## 5 Discussion

The past decade has witnessed a rapid transition from conventional machine-learning pipelines to end-to-end deep networks and, most recently, to transformer-enhanced architectures for pancreatic cancer. Early studies relied on hand-crafted radiomic and clinical variables coupled with support-vector machines, random forests or shallow neural networks. These models provided useful proof-of-concepts but were limited by the size and heterogeneity of available datasets and required expert feature engineering (22, 26). Deep-learning approaches alleviated some of these constraints by learning hierarchical features directly from images. Modified VGG and ResNet models achieved internal accuracies around 98%–99% for binary detection when trained on balanced CT or histology datasets (46, 48). Hybrid stacks that combined denoising, segmentation and deep belief networks further pushed sensitivity to nearly 100% on curated cohorts (48). More recent work incorporates multi-dimensional attention and transformer components to better capture long-range context and focus the network on the small, heterogeneous pancreas and tumor region. For instance, the Multi-Dimensional Attention Gate network (MDAG-Net) introduces spatial, channel and multi-input attention gates; compared with a U-Net baseline on the Task07 Pancreas dataset it improved Dice, precision, recall and mean IoU by 5.3, 1.5, 12.7, and 7.6 percentage points, respectively. This shift illustrates how attention helps filter redundant background features and recalibrates convolution kernels to emphasize tumor regions.

Progress has been driven not only by algorithmic innovation but also by access to larger and more diverse datasets. Early efforts were restricted to two-dimensional slices or small, single-center cohorts; for example, the PCCD dataset comprised 1,800 portal-venous slices, and several urine-biomarker studies contained fewer than 600 samples. Contemporary studies leverage full three-dimensional volumes and multi-center registries. Large-scale cohorts such as PANDA aggregated more than 3,000 non-contrast CTs with external validation across nine hospitals, enabling robust training and external assessment (15). Public benchmarks like the NIH, MSD Task07, and Synapse datasets now provide standardized segmentation tasks, fostering fair comparison and transfer learning. Meanwhile, new modalities—fundus photography, plasma-omics, and multi-phase CT fused with clinical biomarkers—have diversified the data landscape (18, 79). This variety has underscored the importance of modality-specific architectures: two-dimensional patch CNNs remain effective for dense histology or fundus imagery, whereas three-dimensional U-Nets and transformers excel on volumetric CT, and attention-based fusion layers integrate imaging with lab tests or clinical variables.

The model-summary (Tables 5–7) and the data-source (Table 8) together highlight a consistent pattern: (i) radiomics and conventional ML are strongest for prognosis and longitudinal monitoring, where repeated measures (delta-radiomics on serial CT or pre/post-RT PET/CT) capture treatment-induced change and heterogeneity (26–28); (ii) CNNs dominate lesion detection/segmentation on volumetric contrast CT—especially when anatomical context (pancreas/duct masks, secondary signs) is injected—yielding high AUROC with clinically interpretable by-products (masks, heat-maps) (56, 57); and (iii) transformer/attention components are most compelling when fusing modalities (dual-phase CT with laboratory values, or imaging with clinical covariates) and when global context matters (e.g., multi-center non-contrast CT) (15, 75, 77). In short, the “what each method is good at” in Tables 5–7 maps directly to the data regimes cataloged in Table 8: hand-engineered features plus survival models for serial/prognostic settings; CNNs for voxel-level localization; and attention/transformers for multi-modal fusion and long-range dependencies.

Recent transformer-enhanced models demonstrate state-of-the-art performance on both detection and segmentation tasks. Hybrid DenseNet models incorporating channel and spatial attention and eleven clinical variables achieved 92.44% accuracy, AUC 0.971 and F1-score 0.936 for differentiating serous vs. mucinous cystic neoplasms on MRI. The dual-attention TransUNet (DA-TransUNet) integrates positional and channel attention blocks into a U-shaped transformer; on the multi-organ Synapse dataset it improved mean Dice by 2.32 percentage points and reduced the Hausdorff distance by 8.21 mm relative to TransUNet, with a pancreas-specific Dice gain of 5.73 percentage points. Such gains come at minimal computational cost—only a 2.54% increase in parameters. Similarly, the PancreasNet progressive residual transformer network combines Swin blocks, enhanced feature reweighting and regulated fusion; it reported 92.4% accuracy, 93.1% recall, 90.7% specificity, and Dice 0.87 on a 290-volume cohort, outperforming earlier convolutional CAD systems by 5–7 percentage points. Multi-modal attention further enhances classification: a dual-phase CT plus clinical biomarker model achieved 80% accuracy, 0.82 precision, 0.86 specificity, 0.74 recall, and AUC 0.83 for pre-operative lymph-node metastasis prediction, representing improvements of 0.10–0.16 points over radiomics baselines. At the extreme, the DeepOptimalNet cascade achieved a reported 99.3% accuracy, 99.1% sensitivity, and 99.5% specificity for pancreatic tumor classification in CT imaging (83), though such perfect scores on limited datasets warrant cautious interpretation and external validation. Collectively, these results indicate that attention-augmented architectures provide tangible benefits in focusing on subtle pancreatic features, balancing precision and recall, and reducing false positives.

To provide a comprehensive quantitative overview, Table 9 aggregates reported performance metrics across the representative studies in Tables 5–7. Across conventional machine-learning approaches, AUC values ranged from 0.84 to 0.98, with accuracy spanning 81%–99.97%; deep-learning models improved these ranges to AUC 0.92–0.99 and accuracy 82.5%–99.8%, while also introducing segmentation capabilities (Dice 0.19–0.70). Attention and transformer-based architectures achieved the highest overall discrimination [AUC up to 0.996 for PANDA (15)] and best segmentation performance [Dice 0.87 for PancreasNet (74)], with F1 scores consistently exceeding 0.92 across all generations and peaking at 0.97 for the kernel attention network (65). This progression demonstrates measurable performance gains from conventional ML through deep learning to attention/transformer architectures, particularly for complex imaging tasks requiring spatial reasoning and multimodal data fusion. However, it is important to note that these metrics reflect heterogeneous datasets, tasks (detection vs. segmentation vs. prognosis) and validation strategies; therefore, direct cross-study comparisons must account for differences in sample size, prevalence, imaging protocols and patient-level vs. slice-level evaluation.

**Table 9:** Quantitative performance aggregation across AI model generations.

**Metric**	**Conventional ML**	**Deep learning**	**Attention/transformer**	**Best overall**
**AUC**	0.84–0.98	0.92–0.99	0.83–0.996	0.996 [PANDA (15)]
**Dice**	N/A^*a*^	0.19–0.70	0.57–0.87	0.87 [PancreasNet (74)]
**F1**	>0.92^*b*^	0.92–0.96	0.936–0.97	0.97 [KAN (65)]
**Accuracy**	81%–99.97%	82.5%–99.8%	80%–94.4%	99.97% [Decision Tree (21)]

^*a*^ML models focused on classification tasks without segmentation.

^*b*^Exact F1 value not reported; described as “highest” among compared models (20).

Ranges reflect heterogeneous datasets, tasks, and validation strategies; direct cross-study comparisons should account for these differences. AUC, Area Under ROC Curve; N/A, Not Applicable.

A few entries in Tables 5–7 (and related reports) claim near-perfect performance—e.g., 99.8% for a DCNN+DBN pipeline on the 1,800-slice PCCD set (48) and 99.6% for an NASNet → Elman hybrid on a 500-image two-class CT dataset (47). Based on the dataset properties summarized in Table 8 and the authors' own descriptions, several factors likely contribute.

First, both studies used relatively small, balanced research sets (hundreds to low thousands of *images*, not *patients*), often from one or few scanners/centers. Such homogeneity reduces nuisance variation and can inflate apparent performance. Second, PCCD and similar corpora are 2D slice collections where many slices from the *same* patient share textures, field-of-view and reconstruction kernels. If slices (or overlapping patches) from a given patient appear in both train and test folds, leakage occurs, and models learn patient/scanner signatures rather than disease biology, driving metrics toward 99%–100%. Third, several pipelines framed a binary “tumor vs. healthy” task with tumor-centric crops or *post-hoc* ROI selection, simplifying the decision surface and limiting challenging negatives (e.g., pancreatitis, cysts, post-operative change). Finally, neither study reports robust, multi-center external testing at the *patient* level; metrics were typically computed on balanced sets and may not reflect screening prevalence or case-mix.

These considerations help explain why 99%+ results are rare in larger, multi-institutional cohorts or prospective settings. In line with the trends in Table 8, when studies scale to diverse volumes and enforce patient-level splits with external validation, reported AUROC/accuracy typically settle into the 0.83–0.96 range for CAD, and Dice for pancreas/tumor segmentation into the 0.57–0.88 range—credible but not “perfect” (56, 69, 82).

To close the gap between Tables 5–8 and clinical reality, we recommend that future work:

1. Enforce **patient-level** train/validation/test segregation (no slice/patch overlap),
2. report both *image-level* and *patient-level* metrics with **case-mix** and **prevalence** made explicit,
3. Include **external, multi-center** testing and, when possible, **prospective** evaluation,
4. Stratify results by lesion size (< 2 cm), stage, and scanner/protocol,
5. Provide **calibration** (reliability) curves and decision-curve analysis in addition to accuracy/AUC, and
6. Release preprocessing code and split manifests to mitigate inadvertent leakage.

Where feasible, anatomically informed attention (ducts, vessels) and multi-modal fusion should be prioritized for tasks that intrinsically require global context (e.g., lymph-node metastasis or subtype attribution) (15, 77).

Table 3 presents a comprehensive methodological quality assessment across 24 studies from three model generations (Machine Learning, Deep Learning, and Attention/Transformer architectures), systematically analyzing single-center vs. multi-center designs and internal vs. external validation strategies—key quality indicators that directly impact model generalizability and clinical translation.

The evolution of AI models has increasingly emphasized holistic patient context. Sex-specific RNA-Seq survival predictors illustrate how biological sex influences transcriptomic signatures and survival modeling (18). Multi-omics panels combining proteins and gene expression have outperformed single-omics classifiers, highlighting the synergy of heterogeneous data sources (20). The lymph-node metastasis study cited above fused dual-phase CT and eleven laboratory variables via an attention-based fusion module; this integration raised AUC from 0.72 (radiomics alone) to 0.83. Similar improvements are observed when fusing imaging with risk factors: logistic regression on urine biomarkers plus CA19-9 improved sensitivity and specificity to 96%, and multi-head fusion of dual-phase CT with clinical biomarkers increased lymph-node detection accuracy by over 10 percentage points. Beyond imaging, end-to-end frameworks now incorporate self-supervision and pseudo-lesion pretext tasks to reduce annotation burdens; models pre-trained on synthetic lesions achieved external accuracies up to 82.5% despite using only 10% of labeled data (60).

## 6 Emerging directions and future challenges

Although remarkable progress has been made, several challenges remain. Many studies are retrospective and single-center; prospective, multi-institutional trials are needed to verify generalisability and clinical impact. Domain shift across scanners, patient populations and acquisition protocols can degrade performance; attention-based causal regularization and uncertainty estimation may mitigate such shifts. Interpretation and fairness are critical: anatomically guided attention and uncertainty-aware mechanisms provide more transparent saliency maps and allow models to defer to clinicians when confidence is low. In addition, counterfactual explanations should be incorporated to further enhance interpretability and strengthen clinician trust. Early detection remains an open frontier. Delta-radiomics and longitudinal EHR models have shown that subtle texture changes or clinical patterns can predict pancreatic cancer 24 months before diagnosis (22). Recent work suggests that radiomic signatures in apparently normal pancreas can precede diagnosis by over a year. Translating such findings into practice will require precise volumetric segmentation and integration with high-risk cohort selection. Combining imaging with genomics, proteomics, and metabolomics may uncover prodromal signatures, while non-invasive modalities such as fundus photography already hint at systemic biomarkers. Finally, data privacy, algorithmic bias and the integration of AI outputs into clinical workflows require careful consideration. The next decade will likely see the convergence of self-supervised learning, causal graph modeling, multi-modal fusion and federated training to deliver interpretable and equitable AI tools for the early detection, staging, and management of pancreatic cancer. To ensure clinical translation, future studies must also adopt prospective, population-based trial designs that directly evaluate real-world screening and triage performance.

Among the surveyed studies, **PANDA** (9-center, 3,208 training CTs, 5,337 external validation cases, AUC 0.984–0.987) (15) and **Chen et al.'s nationwide study** (1,473 real-world CTs, AUC 0.95, 89.7% AI sensitivity vs. 74.7% radiologist for < 2 cm tumors) (82) represent the closest candidates for clinical deployment, distinguished by multi-center validation and head-to-head clinician comparisons. Beyond imaging, **Nasief's delta-radiomics** (treatment response prediction, external AUC 0.98) (26) and **biomarker panels** [PancRISK urine test, AUC 0.94 (32); plasma amino-acids, AUC 0.86 (33)] show strong translational potential through interpretability and integration with existing workflows. However, regulatory approval faces critical barriers: 83.3% of studies lack multi-center validation required by FDA/EMA for generalizability evidence (Table 3); only four studies documented prospective reader comparisons mandated for Software as a Medical Device (SaMD) submissions; and domain shift effects [11.8% performance drop in cross-ethnic validation (60)] highlight inadequate robustness testing. The EU AI Act (2024) further mandates algorithmic transparency, bias audits across patient subgroups, and uncertainty quantification—requirements met by < 5% of reviewed studies.

### 6.1 Data-sharing frameworks are critical yet underdeveloped accelerators of translation

Public benchmarks (Medical Segmentation Decathlon, NIH pancreas dataset) enable algorithmic comparison but lack patient metadata for robust generalizability assessment (68, 69). Federated learning—where models train locally while sharing only gradient updates—offers privacy-preserving multi-center collaboration, yet zero reviewed studies employed this approach. International consortia (TCIA, PANDA, AI-PREDICT) demonstrate feasibility of de-identified data pooling with harmonized protocols, while standardized reporting (TRIPOD-AI, STARD-AI) remains non-mandatory despite regulatory necessity. Accelerating translation requires: (1) multi-center designs as the methodological standard (currently only 16.7%); (2) federated learning adoption to overcome privacy barriers; (3) expanded data-sharing consortia with incentivized participation; and (4) prospective trials with locked algorithms and independent test sets to satisfy FDA 510(k) evidentiary requirements. Without these infrastructure investments, even high-performing AI risks remaining research curiosities rather than deployed clinical tools.

## 7 Conclusion

This review has traced the methodological progression from classical machine-learning and radiomics to deep representation learning and, most recently, attention- and transformer-enhanced architectures for pancreatic cancer detection, segmentation and prognosis. Attention-augmented models and multi-modal fusion have delivered consistent gains in diagnostic accuracy, segmentation Dice and AUC across a range of tasks and datasets, demonstrating clear potential to augment human readers and to enable earlier, non-invasive detection.

Despite these advances, several barriers remain before routine clinical deployment is possible. Many published studies are retrospective, single-center or underpowered; evaluation metrics and reporting are heterogeneous; and model robustness to domain shift, scanner variability and population differences is often insufficiently tested. Moreover, challenges around interpretability, fairness, data governance and workflow integration persist and must be addressed alongside pure algorithmic improvements.

To accelerate safe and equitable translation we recommend the following priorities:

- Move beyond retrospective benchmarks by testing models in prospective cohorts and pragmatic clinical settings to quantify real-world benefit and harms.
- Adopt common reporting standards, share pre-processing pipelines and encourage public, well-annotated multi-center datasets to reduce evaluation heterogeneity.
- Invest in methods for domain generalization, calibration, uncertainty estimation and causal regularization to reduce sensitivity to scanner/protocol and population shifts.
- Prioritize architectures that fuse imaging with biomarkers and clinical data while remaining computationally efficient and interpretable for deployment.
- Use federated learning and secure model-sharing to scale training data while respecting patient privacy and jurisdictional constraints.
- Co-design algorithms with clinicians, evaluate decision-impact (not just performance metrics), and plan regulatory, ethical and cost-effectiveness assessments early in development.

In summary, the combination of anatomically guided attention, principled multi-modal fusion, and rigorous external validation offers the most promising path toward clinically useful AI for pancreatic cancer. If future work couples these technical advances with prospective trials, clear reporting standards and careful attention to fairness and deployment, AI could materially improve early detection, staging and personalized management for patients with pancreatic cancer.

## Data Availability

The original contributions presented in the study are included in the article/supplementary material, further inquiries can be directed to the corresponding author.
